# Anoctamin 4 channel currents activate glucose-inhibited neurons in the mouse ventromedial hypothalamus during hypoglycemia

**DOI:** 10.1172/JCI163391

**Published:** 2023-07-17

**Authors:** Longlong Tu, Jonathan C. Bean, Yang He, Hailan Liu, Meng Yu, Hesong Liu, Nan Zhang, Na Yin, Junying Han, Nikolas A. Scarcelli, Kristine M. Conde, Mengjie Wang, Yongxiang Li, Bing Feng, Peiyu Gao, Zhao-Lin Cai, Makoto Fukuda, Mingshan Xue, Qingchun Tong, Yongjie Yang, Lan Liao, Jianming Xu, Chunmei Wang, Yanlin He, Yong Xu

**Affiliations:** 1 Children’s Nutrition Research Center, Department of Pediatrics, Baylor College of Medicine, Houston, Texas, USA.; 2 Brain glycemic and metabolism control department, Pennington Biomedical Research Center, Louisiana State University, Baton Rouge, Louisiana, USA.; 3 Department of Neuroscience, Baylor College of Medicine, Houston, Texas, USA.; 4 Department of Molecular and Human Genetics, Baylor College of Medicine, Houston, Texas, USA.; 5 The Cain Foundation Laboratories, Jan and Dan Duncan Neurological Research Institute at Texas Children’s Hospital, Houston, Texas, USA.; 6 Brown Foundation Institute of Molecular Medicine, University of Texas Health Science Center at Houston, Houston, Texas, USA.; 7 Department of Molecular and Cellular Biology, Baylor College of Medicine, Houston, Texas, USA.

**Keywords:** Neuroscience, Diabetes, Glucose metabolism

## Abstract

Glucose is the basic fuel essential for maintenance of viability and functionality of all cells. However, some neurons — namely, glucose-inhibited (GI) neurons — paradoxically increase their firing activity in low-glucose conditions and decrease that activity in high-glucose conditions. The ionic mechanisms mediating electric responses of GI neurons to glucose fluctuations remain unclear. Here, we showed that currents mediated by the anoctamin 4 (Ano4) channel are only detected in GI neurons in the ventromedial hypothalamic nucleus (VMH) and are functionally required for their activation in response to low glucose. Genetic disruption of the *Ano4* gene in VMH neurons reduced blood glucose and impaired counterregulatory responses during hypoglycemia in mice. Activation of VMH^Ano4^ neurons increased food intake and blood glucose, while chronic inhibition of VMH^Ano4^ neurons ameliorated hyperglycemia in a type 1 diabetic mouse model. Finally, we showed that VMH^Ano4^ neurons represent a unique orexigenic VMH population and transmit a positive valence, while stimulation of neurons that do not express Ano4 in the VMH (VMH^non-Ano4^) suppress feeding and transmit a negative valence. Together, our results indicate that the Ano4 channel and VMH^Ano4^ neurons are potential therapeutic targets for human diseases with abnormal feeding behavior or glucose imbalance.

## Graphical abstract

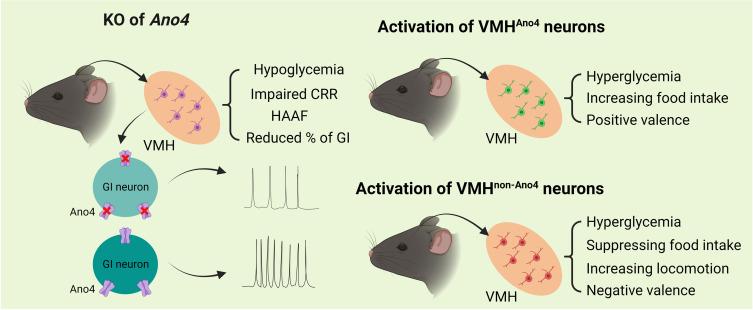

## Introduction

Glucose provides the most essential fuel source for the brain. Glucose levels, therefore, need to be tightly regulated and maintained within a narrow physiological range. Cumulative evidence suggests that the brain is of paramount importance for glucose sensing and whole-body glucose homeostasis (1–3). There are a large number of glucose-sensing neurons in the brain (4, 5) that detect glucose fluctuations and coordinate appropriate endocrine and behavioral responses to restore euglycemia (6, 7).

The hypothalamus, particularly the ventromedial hypothalamic nucleus (VMH), has been recognized as one of the most critical brain regions for glucose sensing and whole-body glucose homeostasis (8, 9). Early studies have shown that lesions of the VMH result in obesity and blunted counterregulatory responses to insulin-induced hypoglycemia (10, 11). Local glucopenia induced by infusion of 2-deoxy-D-glucose (2-DG, a glucose metabolism blocker) into the VMH increases plasma glucagon levels, concomitant with an elevated level of circulating glucose. By contrast, infusion of glucose directly into the VMH reduces glucagon release in the presence of the systemic hypoglycemia (12, 13). Mice with genetic loss of glutamate release from VMH neurons exert a lower fasting glucose level and impaired counterregulatory response to insulin-induced hypoglycemia and glucopenia (14).

The VMH is enriched in glucose-sensing neurons, some being excited by an increase of extracellular glucose levels — referred to as GE neurons — and others being inhibited by high glucose — GI neurons — that can be activated in response to a reduction in extracellular glucose levels (4, 15). Which ion channels are responsible for mediating opposite electric responses in GE and GI neurons upon exposure to the same glucose fluctuations remains a fundamental question. The ion channels mediating GE sensing have been extensively investigated. High glucose leads to an increase of the ATP-ADP ratio and the closure of the ATP-sensitive potassium (K_ATP_) channels. This leads to depolarization of resting membrane potential and calcium entry through the voltage-gated calcium channels to increase neuronal activity (8, 16). Compared with this well-established ionic mechanism for the VMH GE sensing, the ion channels that mediate responses of VMH GI neurons, on the other hand, remain unknown (17).

Another open question is how GE versus GI neurons can be neurochemically distinguished. The answer to this question would be of great value for basic and translational research targeting these populations. A vast majority of VMH neurons can be exclusively labeled by steroidogenic factor 1 (SF1) (18, 19). It has been demonstrated that optogenetic stimulation of VMH^SF1^ neurons increases blood glucose, whereas others reported that chemogenetic activation of VMH^SF1^ neurons decreases blood glucose during glucose tolerance tests (GTTs) and insulin tolerance tests (ITTs) (20, 21). The opposite outcomes of these early studies using SF1 to target VMH neurons may reflect the complex heterogeneity of the VMH SF1 population, which contains both GE and GI neurons, as well as those that cannot sense glucose. Recent research efforts have started to reveal various VMH markers for distinct glucose-regulatory functions. For instance, a subset of VMH GE neurons that express melanocortin 3 receptor (MC3R) can be inhibited by low glucose but excited by high glucose, and activation of these GE VMH^MC3R^ neurons blunts glucose excursion during a GTT (22). On the other hand, a subset of VMH neurons expressing pituitary adenylate cyclase-activating peptide (PACAP) can be activated by low glucose. Importantly, activation of VMH^PACAP^ neurons increases blood glucose, suggesting a GI property of these VMH^PACAP^ neurons (23). Similarly, activation of VMH neurons expressing glucokinase (GcK) (24), nitric oxide synthase 1 (NOS1) (25) and cholecystokinin B receptor (CCKBR) (26), also increases blood glucose, suggesting GcK, NOS1, and CCKBR as putative GI markers as well. However, it remains unclear whether these putative GI markers identify the same or partially overlapping VMH subpopulations. A more important question is whether the majority of VMH GI neurons can be identified by a common neurochemical marker (17).

In the present study, we first utilized published single-cell RNA-Seq data from mouse and macaque VMH to reveal that anoctamin 4 (Ano4, a calcium-activated chloride channel protein) shows the highest overlapping pattern with putative VMH GI markers. We then used electrophysiology to demonstrate that Ano4-mediated currents exclusively exist in VMH GI neurons and that these currents mediate activation of VMH GI neurons induced by low glucose. Using mice lacking Ano4 in the VMH, we further examined the physiological functions of Ano4 channel in the regulation of energy and glucose homeostasis. Finally, we generated a novel Ano4-P2A-Cre knock-in mouse model and combined chemogenetic, optogenetic (both Cre-on and Cre-off), and Kir2.1-mediated chronic inhibition strategies to reveal segregated functions of VMH^Ano4^ neurons versus. VMH^non–Ano4^ neurons.

## Results

### Ano4 mediates glucose sensing of VMH GI neurons.

**Figure 1:**
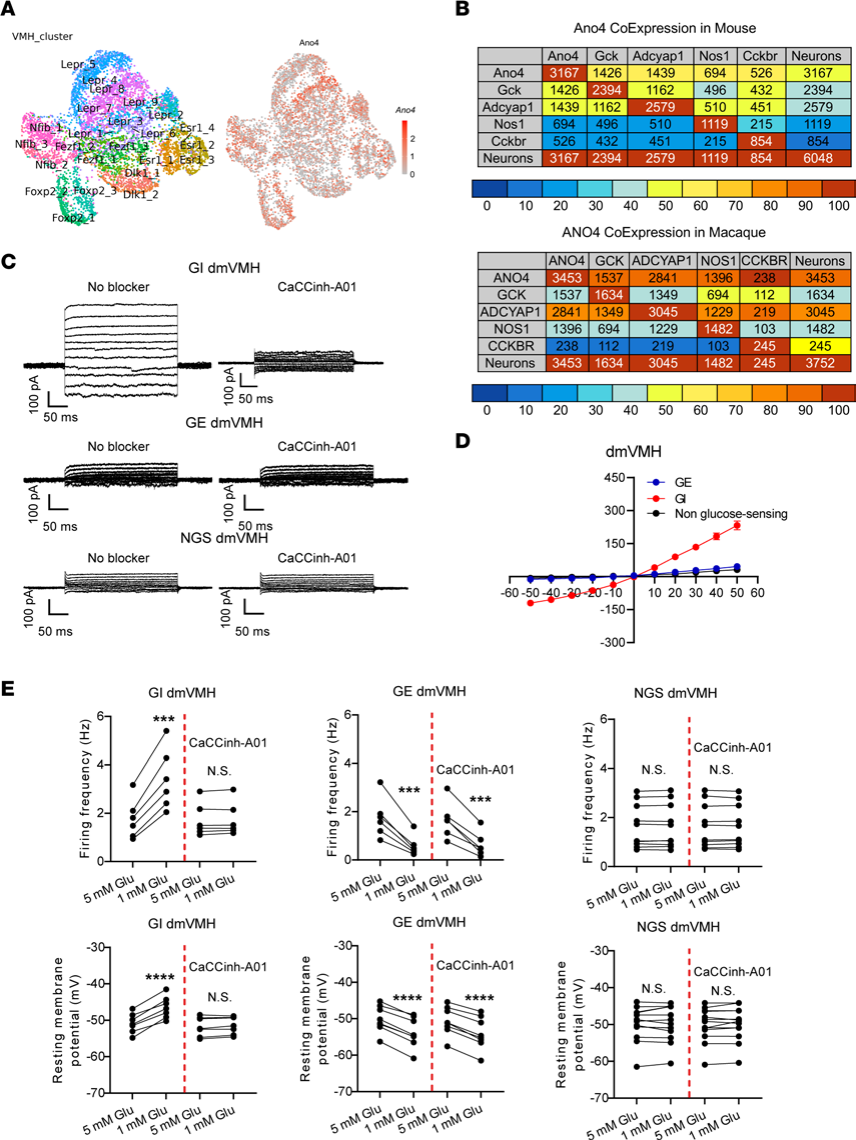
Ano4 mediates glucose sensing of VMH GI neurons. (**A**) Clusters classified as VMH neurons mapped in Uniform Manifold Approximation and Projection (UMAP) space and Ano4 expression in VMH neurons mapped in same UMAP space. (**B**) Heatmaps for mouse and macaque, where numbers in table-cells represent the number of neurons that coexpress the gene from the row, and colors represent the percentage of neurons coexpressing the gene from the row out of the total number of neurons expressing the gene from the column. (**C**) Typical traces of Ano currents in the dmVMH neurons in the absence or the presence of an Ano inhibitor CaCCinh-A01. (**D**) Ano current was detected in GI neurons in the dmVMH, but not in GE or NGS neurons (*n* = 6 for GE, *n* = 5 for GI, and *n* = 6 for NGS). (**E**) Firing frequency and resting membrane potential of GI, GE, and NGS neurons in the dmVMH, under glucose exposure from 5 → 1 mM in the absence or the presence of an Ano inhibitor CaCCinh-A01 (*n* = 8 for GE, *n* = 6 for GI, and *n* = 10 for NGS). Data are expressed as mean ± SEM. Significant differences between 5 mM glucose and 1 mM glucose are shown as ***P < 0.001 and ****P < 0.0001 in a paired 2-tailed *t* test (**E**).

We have previously demonstrated that Ano4, a chloride channel, mediates hypoglycemia-induced activation in ERα-expressing GI neurons in the ventrolateral subdivision of the VMH (vlVMH) (27). Here we further explored the possibility that Ano4 provides a common mechanism for GI glucose sensing in the entire VMH. To this end, we first performed the secondary analysis of single-cell RNA-Seq data from the mouse VMH (28). Interestingly, the distribution of *Ano4* was not restricted to the vlVMH, but was in the whole VMH (Figure 1A). We then analyzed the overlapping pattern of *Ano4* with other putative GI markers — i.e., *Gck*, *Adcyap1*, *Nos1*, and *Cckbr* — within the mouse VMH neurons (Supplemental Figure 1; supplemental material available online with this article; https://doi.org/10.1172/JCI163391DS1). These putative GI markers only showed 16.6%–52.8% mutual overlapping among themselves — *Gck* 45.1%–50.9%, *Adcyap*1 44.4%–52.8%, *Nos1* 19.8%–32.7%, and *Cckbr* 16.6%–19.2% — indicating that each of these markers only labels a subpopulation of VMH GI neurons. On the other hand, *Ano4* is coexpressed by 55.8%–62.0% of neurons labeled by any of these markers: *Gck* 59.6%, *Adcyap1* 55.8%, *Nos1* 62.0%, and *Cckbr* 61.59% (Figure 1B). Importantly, the single-cell RNA-Seq data from the macaque VMH (28) revealed a more than 90% coexpression of *ANO4* with other putative GI markers (Figure 1B). These results raised the possibility that Ano4 is a generic marker for a large portion of GI neurons in the VMH.

To further test this possibility, we used slice electrophysiology to explore a potential association between the glucose-sensing properties and Ano4-mediated currents in randomly selected neurons across the entire VMH, including the dorsomedial VMH (dmVMH), the central VMH (cVMH) and the vlVMH (Supplemental Figure 2A). As we reported before (27), the glucose-sensing properties of VMH neurons were examined under the current-clamp mode in response to a 5 → 1 mM extracellular glucose fluctuation protocol (see Methods). Among the tested dmVMH neurons, we found that 28.6% were GE neurons (≥ 2 mV hyperpolarized by 5 → 1 mM glucose fluctuation), 25.0% were GI neurons (≥ 2 mV depolarized by 5 → 1 mM glucose fluctuation) and 46.4% were nonglucose-sensing (NGS) neurons (< 2 mV changes in resting membrane potential by 5 → 1 mM glucose fluctuation) (Supplemental Figure 2, B and C). Similarly, the percentages of GE/GI/NGS neurons in the cVMH were 25.9%, 33.3%, and 40.8%, respectively, and were 34.6%, 26.9% and 38.5% in the vlVMH, respectively (Supplemental Figure 2, B and C). Importantly, GI neurons in all VMH subdivisions (dmVMH, cVMH, or vlVMH) exhibited Ano currents that were abolished by an Ano inhibitor CaCCinh-A01 (Figure 1, C and D and Supplemental Figure 3, A and B). On the other hand, GE and NGS neurons in all subdivisions did not exhibit such currents (Figure 1, C and D and Supplemental Figure 3, A and B). In addition, CaCCinh-A01 treatment blocked hypoglycemia-induced increases in firing frequency and membrane potential in GI neurons from all VMH subdivisions, whereas CaCCinh-A01 did not affect responses in GE neurons or NGS neurons (Figure 1E and Supplemental Figure 3, C and D). Moreover, we repeated the same electrophysiological recordings in the presence of synaptic blockers (TTX, CNQX, D-AP5, and bicuculline), and found that the antagonistic effect of CaCCinh-A01 still existed in GI neurons in all subdivisions, indicating that the effect was independent of synaptic inputs (Supplemental Figure 4, A–D). Together, these results indicate that Ano currents are exclusively present in GI neurons across the entire VMH and are required to mediate their activation in response to low glucose.

### Loss of Ano4 in VMH neurons impairs glucose balance.

**Figure 2:**
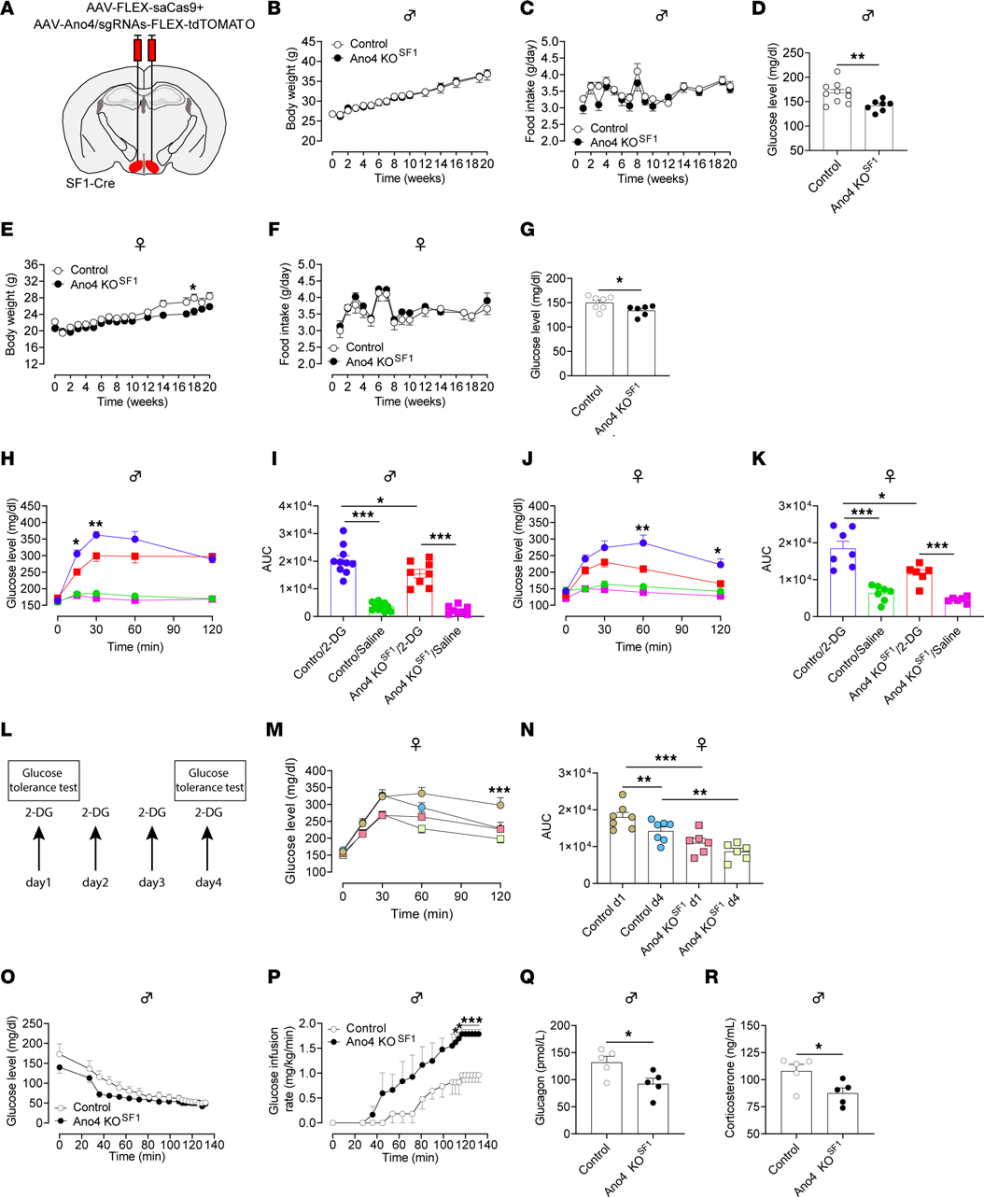
Knockout of *Ano4* in VMH^SF1^ neurons induces hypoglycemia and blunts counterregulatory response. (A) Schematic representation of CRISPR-mediated knockout of *Ano4* in both sides of VMH^SF1^ neurons in SF1-Cre mice. (B–D) Body weight, food intake, and blood glucose in male mice (*n* = 10 for control, and *n* = 7 for Ano4 KO^SF1^). (E–G) Body weight, food intake, and blood glucose in female mice (*n* = 10 for control, and *n* = 7 for Ano4 KO^SF1^). (H–K) Blood glucose levels and respective AUC after treatment with 2-DG (300 mg/kg, i.p.) or saline (10 mL/kg, i.p.) in male mice (H and I, *n* = 10 for control, and *n* = 7 for Ano4 KO^SF1^) and female mice (J and K, *n* = 7 for control, and *n* = 6 for Ano4 KO^SF1^). (L–N) Response to repeated treatment with 2-DG (300 mg/kg, i.p.) with glucose measurement on day 1 and day 4 in female mice (*n* = 7 for Control, and *n* = 6 for Ano4 KO^SF1^). (O–R) Blood glucose levels (O) and glucose infusion rate (P) throughout the recording of hyperinsulinemic-hypoglycemic clamp in male mice (*n* = 5 for control and Ano4 KO^SF1^). Blood glucagon and corticosterone levels at the fixed hypoglycemic status (Q–R, *n* = 5 for control and Ano4 KO^SF1^). Data are expressed as mean ± SEM. Significant differences between control and Ano4 KO^SF1^ groups are shown as *P < 0.05, **P < 0.01, and ***P < 0.01 (2-tailed Student’s t test for D, G, Q and R; 2-way ANOVA followed by Bonferroni tests for E, H, J, M, and P; 1-way ANOVA with Bonferroni’s adjustment for multiple comparisons for I, K, and N.).

Given the role of VMH neurons in glucose homeostasis and feeding behavior, we sought to examine the physiological functions of Ano4 in the VMH on regulation of glucose and food intake. We employed CRISPR-Cas9 approach to KO the *Ano4* gene in VMH neurons. Briefly, we previously validated 2 sgRNAs that induce indel mutations in exon 4 and exon 11 of the mouse *Ano4* gene and constructed these sgRNAs into an AAV vector followed by Cre-dependent FLEX-tdTOMATO sequence (27). Here, SF1-Cre mice received stereotaxic injections of AAV-FLEX-saCas9 and AAV-Ano4/sgRNAs-FLEX-tdTOMATO into both sides of the VMH to disrupt expression of *Ano4* selectively in VMH^SF1^ neurons (Figure 2A and Supplemental Figure 5). For the purpose of the control, WT mouse littermates received the same viruses, and SF1-Cre mice received AAV-FLEX-GFP and AAV-Ano4/sgRNAs-FLEX-tdTOMATO. Since these 2 control groups showed similar phenotypes (data not shown), we combined them in data analysis. KO of *Ano4* did not alter body weight or food intake in male *Ano4*-KO mice compared with control mice (Figure 2, B and C), but did induce a significant reduction in blood glucose (Figure 2D). Similarly, female mice lacking *Ano4* in VMH neurons showed a modest body weight reduction without significant changes in food intake, but a lower blood glucose levels than control mice (Figure 2, E–G). Moreover, KO of *Ano4* did not affect energy expenditure in male or female mice (Supplemental Figure 6).

Both male and female Ano4-KO^SF1^ mice exhibited similar blood glucose fluctuations during GTT and ITT compared with control mice (Supplemental Figure 7). Interestingly, KO of *Ano4* significantly impaired glucose elevations in response to 2-DG-induced glucopenia in both male (Figure 2, H and I) and female mice (Figure 2, J and K). We then used multiple 2-DG treatments to mimic hypoglycemia-associated autonomic failure (HAAF) (26) in female mice (Figure 2L). A 4-day treatment of 2-DG caused a HHAF phenotype in control mice (Figure 2, M and N). On day 1 and day 4 of the HAAF paradigm, Ano4-KO^SF1^ mice had a consistently lower blood glucose level compared with control mice after treatment with 2-DG (Figure 2, M and N). On day 4, control mice had a lower blood glucose level at 120 minutes after 2-DG administration compared with day 1 (Figure 2, M and N), but there was no significant difference at all time points between day 1 and day 4 in Ano4 KO^SF1^ mice. These data suggest that impaired Ano4 function contributes to development of HAAF (Figure 2, M and N).

Since repeated 2-DG treatment resulted in HAAF, the same treatment was very likely to disrupt glucose homeostasis in other scenarios. Therefore, male mice were subjected to a hyperinsulinemic-hypoglycemic clamp study without exposure to the HAAF paradigm. In a hyperinsulinemic-hypoglycemic clamp study where the blood glucose level was clamped at a hypoglycemic level (approximately 50 mg/dL) (Figure 2O), male Ano4 KO^SF1^ mice required a significantly higher glucose infusion rate, indicating a lower endogenous glucose production and/or increased glucose disappearance compared with control mice (Figure 2P). Consistently, the plasma glucagon and corticosterone levels at the hypoglycemic condition were significantly reduced compared with those in control mice in the hypoglycemia condition (Figure 2, Q and R). Overall, these results indicate that Ano4 in VMH neurons is required to maintain a normal counterregulatory response to hypoglycemia.

Slice electrophysiology was used to characterize the percentages of GE/GI/NGS neurons in SF1 control neurons and Ano4 KO^SF1^ neurons (Supplemental Figure 8A). Compared with SF1 control neurons, the percentage of GI neurons among Ano4 KO^SF1^ neurons was remarkably lower (Supplemental Figure 8, B–G). The percentage of GE neurons was comparable between SF1 control neurons and Ano4 KO^SF1^ neurons (Supplemental Figure 8, B–G). Ano current was only detected in GI neurons in response to low glucose, while loss of *Ano4* impaired this current under glucose fluctuation (Supplemental Figure 8H). These data suggest that Ano4 is indispensable for glucose sensing in GI neurons.

### GI sensing by VMH^Ano4^ neurons.

To further explore the physiological functions of Ano4-expressing neurons, we generated an Ano4-P2A-Cre knock-in mouse line. For visualization of Cre-expressing neurons in the brain, we crossed Ano4-P2A-Cre mice with Rosa26-LSL-tdTomato reporter mice to generate Ano4-P2A-Cre/Rosa26-LSL-tdTomato mice (Supplemental Figure 9A). Ano4 neurons from these mice were therefore labeled by tdTomato, as confirmed by dual RNAscope for tdTomato and *Ano4* (Supplemental Figure 9B). Dual RNAscope revealed that about 90% of tdTomato^+^ neurons coexpressed *Ano4*, and about 91.6% *Ano4*-positive neurons coexpressed tdTomato (Supplemental Figure 9, B and C). We found that Ano4 neurons were sparsely distributed in the cVMH and vlVMH, and few were in the dmVMH (Supplemental Figure 9D).

**Figure 3:**
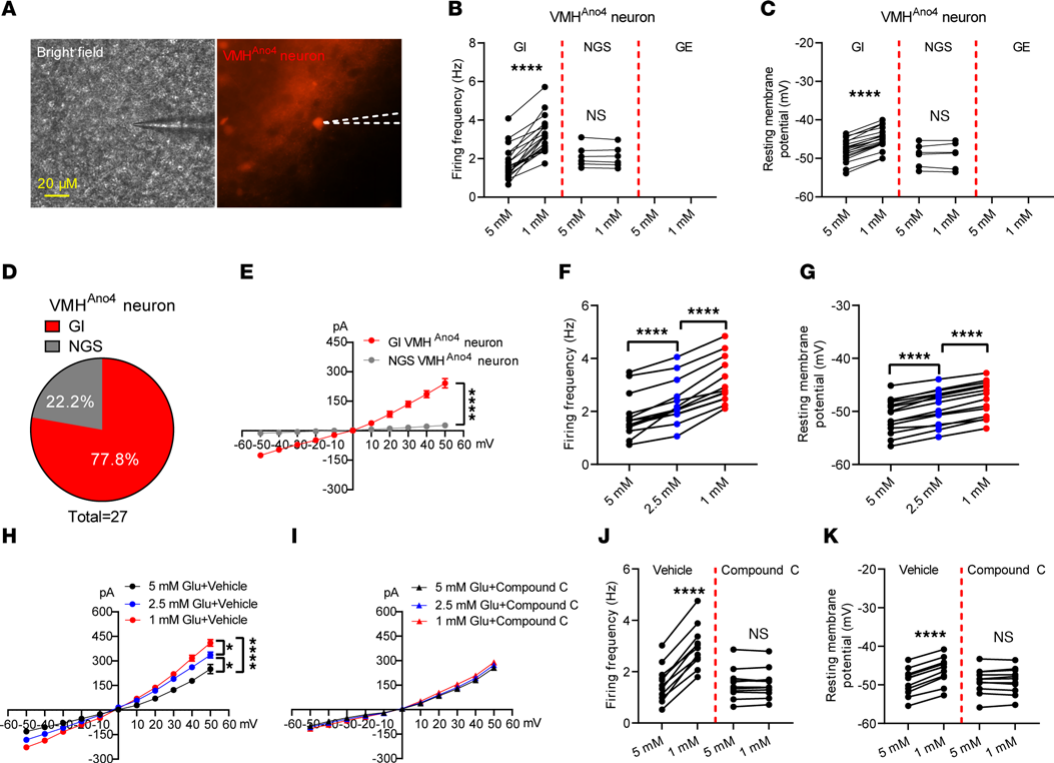
Electrical responses of VMH^Ano4^ neurons to glucose fluctuation. (A) Experimental illustration of a recorded VMH^Ano4^ neuron. (B–D) Firing frequency, resting membrane potential, and percentages of GI and NGS neurons in VMH^Ano4^ neurons under glucose exposure from 5 → 1 mM (*n* = 0 for GE, *n* = 21 for GI, and *n* = 6 for NGS). (E) Ano current was detected in GI VMH^Ano4^ neurons and was minimal in NGS VMH^Ano4^ neurons (*n* = 6 for GI and NGS). (F and G) Firing frequency and resting membrane potential of VMH^Ano4^ neurons under glucose exposure from 5 mM → 2.5 mM → 1 mM (*n* = 13). (H) Ano current detected in VMH^Ano4^ neurons under different glucose fluctuations from 5 mM → 2.5 mM → 1 mM (*n* = 5). (I) Ano current detected in VMH^Ano4^ neurons under different glucose fluctuation was blocked by Compound C (*n* = 5). (J and K) Firing frequency and resting membrane potential of VMH^Ano4^ neurons in response to low glucose in the presence of AMPK blocker Compound C (10 μM) (*n* = 11). Data are expressed as mean ± SEM. Significant differences between 5 mM glucose and 1 mM glucose are shown as ****P < 0.0001 determined by a 2-tailed paired Student’s *t* test for B, C, F, G, J, and K and 2-way ANOVA followed by Bonferroni tests for E and H.

Slice electrophysiology studies further revealed that 77.8% of tdTomato-labeled Ano4 neurons in the VMH (VMH^Ano4^) were GI neurons, and the remaining 22.2% were NGS neurons (Figure 3, A–D). Ano current was detected only in GI VMH^Ano4^ neurons (Figure 3E). To further investigate the mechanisms by which glucose concentrations influence Ano currents, we exposed GI VMH^Ano4^ neurons to a gradient glucose reduction (5 mM → 2.5 mM → 1 mM). We observed dose-dependent increases in firing frequency and resting membrane potential, which was associated with a gradual increase in Ano currents (Figure 3, F–H). It has been reported that AMP-activated protein kinase (AMPK) is involved in glucose sensing of GI neurons (29). We then characterized the effect of an AMPK blocker compound C (10 μM) on Ano currents in response to the 5 mM → 2.5 mM → 1 mM glucose reduction. Interestingly, in the presence of compound C (10 μM), the glucose reductions failed to increase Ano current in GI VMH^Ano4^ neurons (Figure 3I). In addition, compound C blocked increases in firing frequency and resting membrane potential in GI VMH^Ano4^ neurons (Figure 3, J and K).

Importantly, while an i.p. injection of glucose (2 g/kg, i.p.) induced abundant c-fos expression in the VMH, none of the Ano4 neurons expressed c-fos (Supplemental Figure 10, A and B), further confirming that Ano4 does not label GE neurons, but is likely a marker for GI neurons in the VMH. Together, these data suggest that VMH^Ano4^ neurons represent a large portion of GI neurons in the VMH, and low glucose increases the Ano current in GI VMH^Ano4^ neurons via the AMPK pathway.

### Activation of VMH^Ano4^ neurons increases food intake and blood glucose.

**Figure 4:**
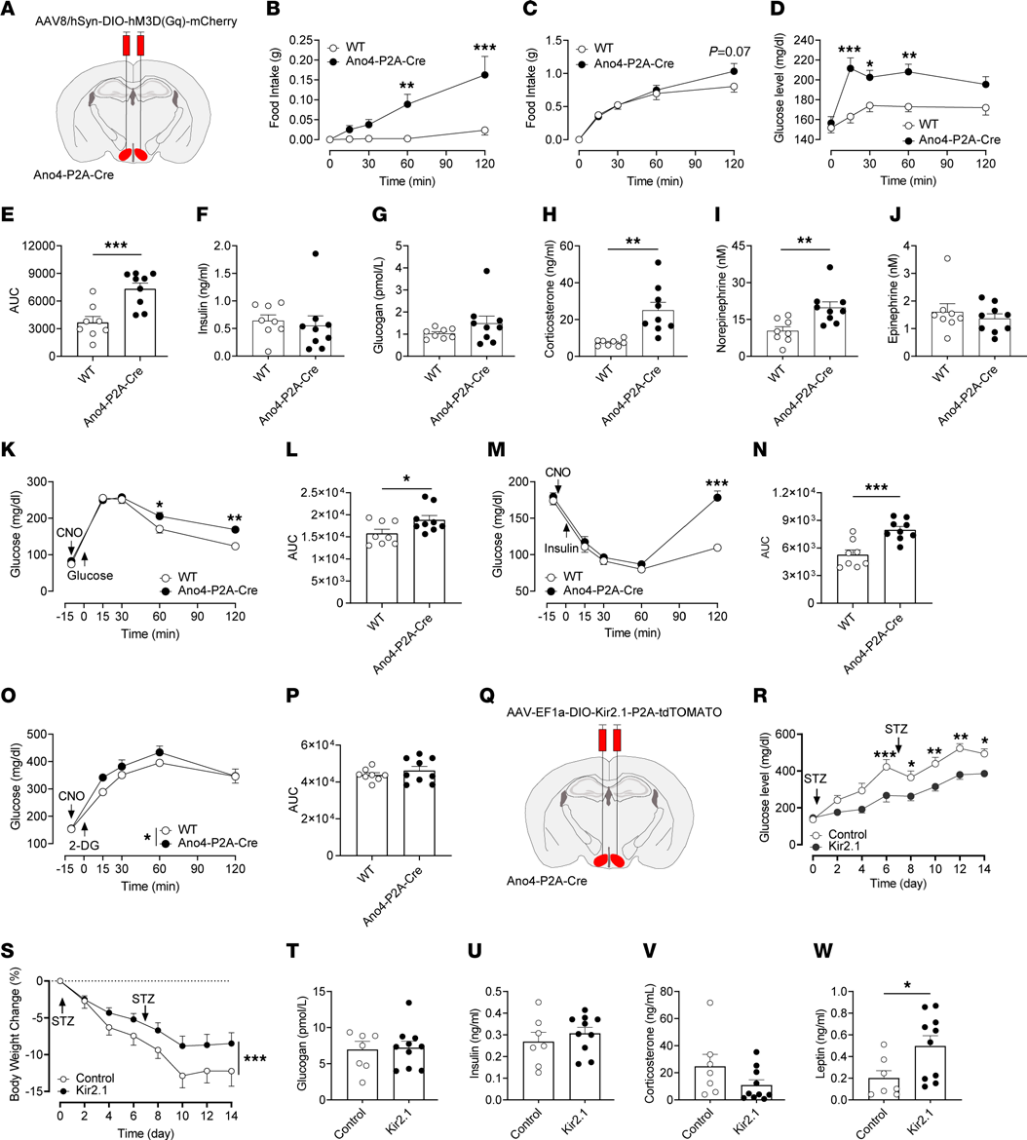
Effects of VMH^Ano4^ neurons on feeding and glycemic control. (A) Schematic diagram of injection of AAV8-hSyn-DIO-hM3D(Gq)-mCherry into the VMH in Ano4-P2A-Cre mice (male, 8–10 weeks of age). (B and C) Food intake in both satiated and fasted condition following activation of VMH^Ano4^ neurons via CNO injection (*n* = 8 for WT, and *n* = 9 for Ano4-P2A-Cre). (D and E) Activation of VMH^Ano4^ neurons elicits hyperglycemia (*n* = 8 for WT, and *n* = 9 for Ano4-P2A-Cre). (F–J) Circulating concentrations of insulin, glucagon, corticosterone, norepinephrine, and epinephrine levels after activation of VMH^Ano4^ neurons (*n* = 8 for WT, and *n* = 9 for Ano4-P2A-Cre). (K and L) Glucose levels during GTT after CNO injection (*n* = 8 for WT, and *n* = 9 for Ano4-P2A-Cre). (M and N) Glucose levels during ITT after CNO injection (*n* = 8 for WT and *n* = 9 for Ano4-P2A-Cre). (O and P) Glucose levels during 2-DG-induced glucopenia after CNO injection (*n* = 8 for WT, and *n* = 9 for Ano4-P2A-Cre). (Q) Schematic diagram of injection of AAV-EF1a-DIO-Kir2.1-P2A-dTOMATO into the VMH in Ano4-P2A-Cre mice. (R and S) Weekly blood glucose and body weight in STZ-treated mice (*n* = 7 for control and *n* = 10 for Kir2.1). (T-W) Blood glucagon, insulin, corticosterone, and leptin levels 2 weeks after STZ treatment (*n* = 7 for control and *n* = 10 for Kir2.1). Data are expressed as mean ± SEM. Significant differences between control and Ano4-P2A-Cre groups are shown as *P < 0.05, **P < 0.01, and ***P < 0.001 (2-tailed Student’s *t* test for E, H, I, L, N, and W, and 2-way ANOVA followed by Bonferroni tests for B, C, D, K, M, O, R, and S).

To test the potential functions of VMH^Ano4^ neurons, we injected AAV8-hSyn-DIO-hM3D(Gq)-mCherry in the VMH of male Ano4-P2A-Cre mice (Figure 4A). VMH^Ano4^ neurons can be activated by clozapine-N-oxide (CNO) (Supplemental Figure 11). WT littermate mice that received the same virus infection and CNO treatment were used as controls. We found that activation of VMH^Ano4^ neurons significantly increased food intake in a satiated condition, and a nonsignificant trend was observed in a fasting condition (Figure 4, B and C). Moreover, when food was not provided in the cages, activation of VMH^Ano4^ neurons significantly increased basal blood glucose levels (Figure 4, D and E). We further showed that chemogenetic activation of VMH^Ano4^ neurons significantly increased corticosterone and norepinephrine levels in the blood, but insulin, glucagon, and epinephrine levels were not affected (Figure 4, F–J). Moreover, increases in blood glucose induced by chemogenetic activation of VMH^Ano4^ neurons were also observed under the conditions of GTT and ITT, and a very mild increase in blood glucose was observed in the scenario of 2-DG treatment (Figure 4, K–P). These results indicate that VMH^Ano4^ neurons are orexigenic and can trigger glucose elevations, both of which are consistent with features from a GI population.

### Chronic inhibition of VMH^Ano4^ neurons attenuates diabetic hyperglycemia.

Because activation of VMH^Ano4^ neurons increased blood glucose, we asked whether inhibition of these neurons would attenuate diabetic hyperglycemia. To this end, we injected AAV-EF1a-DIO-Kir2.1-P2A-dTOMATO (30) into the VMH of male Ano4-P2A-Cre mice (Figure 4Q and Supplemental Figure 12). WT littermate mice that received the same viral infection were used as controls. 4 weeks after surgery, diabetic hyperglycemia was induced by weekly injections of streptozotocin (STZ, 160 mg/kg, i.p.). In control mice, STZ caused stable hyperglycemia (> 450 mg/dL), concomitant with a significant reduction in body weight (approximately 12% maximal loss) (Figure 4, R and S). However, inhibition of Ano4 neurons significantly ameliorated STZ-induced hyperglycemia, as well as body weight loss (Figure 4, R and S). Importantly, the improved diabetic phenotype was independent of pancreatic islet hormones, as there was no significant difference in plasma glucagon or insulin levels (Figure 4, T and U). Moreover, inhibition of VMH^Ano4^ did not affect plasma corticosterone, but significantly increased the leptin level compared with control animals after STZ treatment (Figure 4, V and W). These results indicate that targeting VMH^Ano4^ neurons may represent a therapeutic avenue in glycemic and body-weight control in diabetes.

### VMH^Ano4^ versus VMH^non–Ano4^ neurons differentially regulate food intake, blood glucose, and valence.

**Figure 5:**
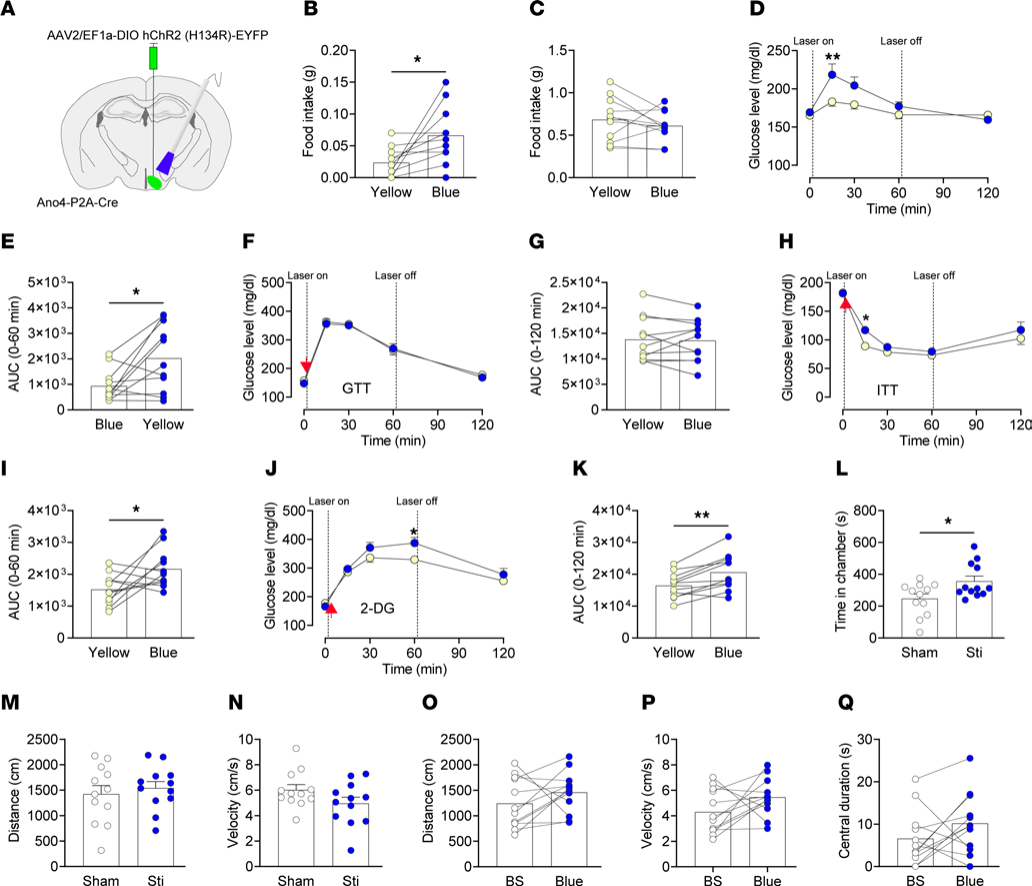
Optogenetic stimulation of VMH^Ano4^ neurons promotes food intake, induces hyperglycemia, and transmits a positive valance. (**A**) Schematic representation of injection of AAV2-EF1a-DIO-hChR2 (H134R)-EFYP into the VMH and implantation of optical fiber in Ano4-P2A-Cre mice (male, 8–12 weeks of age). (**B** and **C**). Effects of optogenetic stimulation of VMH^Ano4^ neurons on food intake in satiated (**B**) and fasted condition (**C**) (*n* = 12). (**D**–**K**) Effects of optogenetic stimulation of VMH^Ano4^ neurons on blood glucose in a basal state (**D** and **E**), in GTT (**F** and **G**), in ITT (**H** and **I**) or during glucopenia induced by 2-DG (**J** and **K**) (*n* = 12). (**L**–**N**) Time spent and distance travelled and velocity in each respective chamber for Ano4-P2A-Cre mice with injection of AAV2-EF1a-DIO-hChR2 (H134R)-EFYP into the VMH during real-time place preference test (*n* = 12). (**O**–**Q**) Distance travelled, velocity, and time spent in the center for Ano4-P2A-Cre mice with injection of AAV2-EF1a-DIO-hChR2 (H134R)-EFYP into the VMH during open field test. BS refers to baseline (*n* = 12). Data are expressed as mean ± SEM. Significant differences between groups are shown as *P < 0.05 and **P < 0.01 determined by 2-way ANOVA followed by Bonferroni tests for **D**, **H** and **J**, 2-tailed paired Student’s *t* test for **B**, **E**, **I** and **K**, and 2-tailed unpaired Student’s t-test for **L**). Red arrows indicate where glucose (**F**), insulin (**H**) or 2-DG (**J**) was injected.

We injected AAV2-EF1a-DIO-hChR2 (H134R)-EFYP in the VMH and implanted optical fibers in male Ano4-P2A-Cre and WT mice (Figure 5A and Supplemental Figure 13A). Channelrhodopsin-2 (ChR2) is a nonspecific cation channel that can be activated by blue light. Optogenetic stimulation of VMH^Ano4^ neurons with blue, but not yellow, light significantly increased food intake in satiated, but not in fasted, conditions (Figure 5, B and C). Furthermore, when food was not provided in the cages, activation of VMH^Ano4^ neurons significantly increased basal blood glucose levels(Figure 5, D and E), as well as during ITT (Figure 5, H and I) and 2-DG-induced glucopenia (Figure 5, J and K), but not during GTT as did ITT (Figure 5, H and I) and 2-DG-induced glucopenia (Figure 5, J and K), but not GTT (Figure 5, F and G). As negative controls, blue or yellow light did not alter food intake or blood glucose in any settings in WT control mice (Supplemental Figure 14).

In the real-time place preference test where optogenetic stimulation of VMH^Ano4^ neurons was timely paired to 1 chamber of a rectangular 2-chamber box, mice spent significantly more time in the stimulatory chamber, without significant changing in distance traveled or velocity (Figure 5, L–N). As controls, yellow light shone onto VMH^Ano4^ neurons did not alter any place preference in the same mice (Supplemental Figure 15, A–C). These data indicate that activation of VMH^Ano4^ neurons transmits a positive valence. In the open field test, optogenetic stimulation of VMH^Ano4^ neurons did not affect distance traveled or velocity compared with baseline (Figure 5, O and P), as well as the time spent in the center area, a measurement of anxiety-like behavior (Figure 5Q). Yellow light shone onto VMH^Ano4^ neurons did not alter locomotor activity or anxiety-like behavior in the same mice (Supplemental Figure 16, A–C). Together, these results indicate that VMH^Ano4^ neurons are orexigenic, increase blood glucose, and transmit a positive valence.

**Figure 6:**
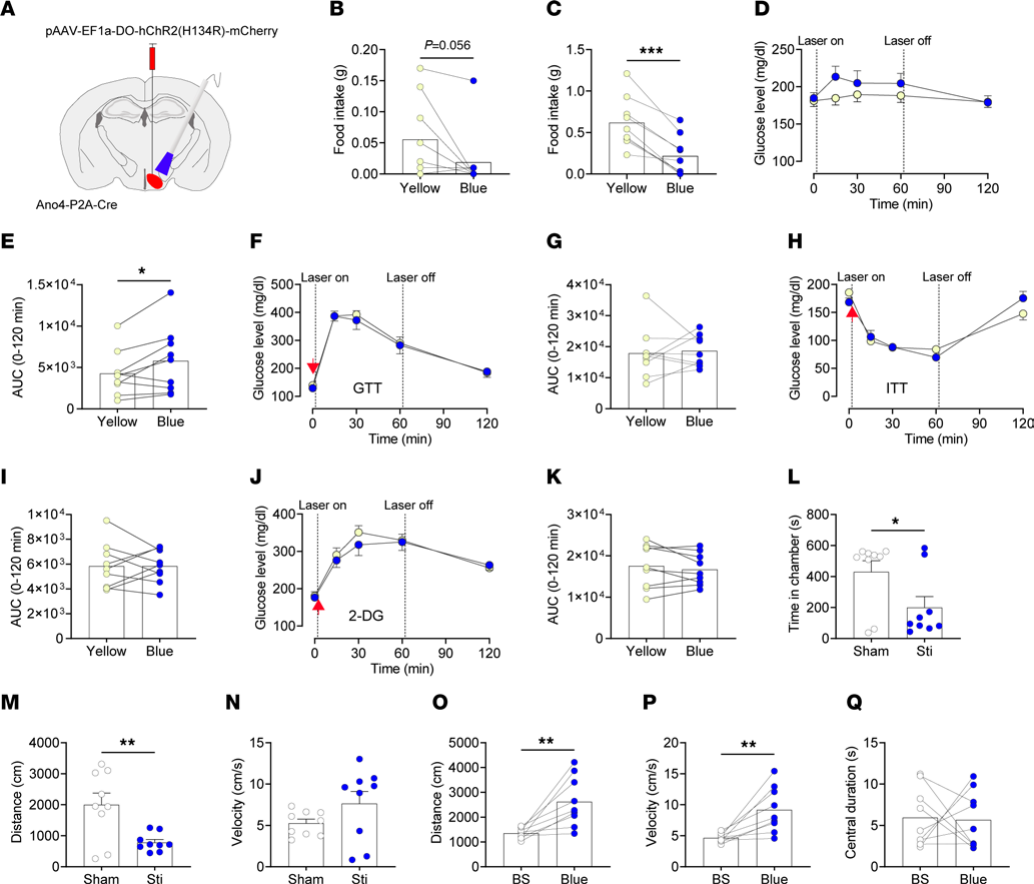
Optogenetic stimulation of VMH^non–Ano4^ neurons suppresses food intake and induces a negative valance. (**A**) Schematic representation of injection of pAAV-EF1a-DO-hChR2 (H134R)-mCherry into the VMH and implantation of optical fiber in Ano4-P2A-Cre mice (male, 8–12 weeks of age). (**B** and **C**) Effects of optogenetic stimulation of VMH^non–Ano4^ neurons on food intake in satiated (**B**) and fasted (**C**) condition mice (*n* = 9). (**D–K**) Effects of optogenetic activation of VMH^non–Ano4^ neurons on blood glucose level in a basal state (**D**–**E**), in GTT(**F** and **G**), in ITT (**H**-**I**), or 2-DG–induced glucopenia (**J**–**K**) (*n* = 9). **L**–**N**. Time spent, distance travelled, and velocity in each respective chamber for Ano4-P2A-Cre mice with injection of pAAV-EF1a-DO-hChR2 (H134R)-mCherry into the VMH during real-time place preference test (*n* = 9). (**O**–**Q**). Distance travelled, velocity, and time spent in the center for Ano4-P2A-Cre mice with injection of pAAV-EF1a-DO-hChR2 (H134R)-mCherry into the VMH during open field test. BS refers to baseline (*n* = 9). Data are expressed as mean ± SEM. Significant differences between groups are shown as *P < 0.05, **P < 0.01, and ***P < 0.001 (2-tailed paired Student’s *t* test for **B**, **C**, **E**, **O**, and **P**, and 2-tailed unpaired Student’s *t* test for **L** and **M**). Red arrows indicate where glucose (**F**), insulin (**H**) or 2-DG (**J**) was injected.

Notably, previous studies reported that activation of VMH^SF1^ neurons reduces food intake (21, 31, 32), which is in opposition to the orexigenic action of VMH^Ano4^ neurons we observed. Thus, we speculated that VMH^Ano4^ neurons represent a unique orexigenic VMH population, while the other VMH neurons that do not express Ano4 are anorexigenic. To test this possibility, we injected a Cre-off optogenetic virus, pAAV-EF1a-DO-hChR2 (H134R)-mCherry (33), into the VMH and implanted optical fiber in male Ano4-P2A-Cre mice (Figure 6A and Supplemental Figure 13B). This virus expressed ChR2 only in neurons without Cre recombinase (VMH^non–Ano4^ neurons). Optogenetic stimulation of VMH^non–Ano4^ neurons trended to inhibit food intake in a satiated condition and significantly suppressed feeding in a fasted condition (Figure 6, B and C). Stimulation of VMH^non–Ano4^ neurons induced a slight increase in blood glucose during a basal state (Figure 6, D and E), but blood glucose responses were not altered during GTT (Figure 6, F and G), ITT (Figure 6, H and I) or 2-DG tests (Figure 6, J and K).

We further examined the effects of VMH^non–Ano4^ neurons on valence and anxiety-like behavior. In the real-time place preference test, mice spent significantly less time in the chamber that was paired with optogenetic stimulation of VMH^non–Ano4^ neurons, which was associated with a significantly shorter traveled distance (Figure 6, L–N). As controls, yellow light shone onto VMH^non–Ano4^ neurons did not alter place preference in the same mice (Supplemental Figure 15, D–F). These data indicate that activation of VMH^non–Ano4^ neurons transmits a negative valence. In the open field test, optogenetic stimulation of VMH^non–Ano4^ neurons significantly increased traveled distance and velocity (Figure 6, O and P), but did not to alter the time spent in the center area (Figure 6Q). Yellow light shone onto VMH^non–Ano4^ neurons did not alter locomotor activity or anxiety-like behavior in the same mice (Supplemental Figure 16, D and E). Together, these results indicate that VMH^non–Ano4^ neurons are anorexigenic and transmit a negative valence.

## Discussion

In the present study, we showed that Ano currents exist specifically in VMH GI neurons and are required to mediate their activation upon hypoglycemia. Genetic disruption of the *Ano4* gene in VMH neurons reduces blood glucose and impairs counterregulatory responses to prevent severe hypoglycemia. Using a newly generated Ano4-P2A-Cre mouse line, we revealed that activation of VMH^Ano4^ neurons increases food intake and blood glucose, while chronic inhibition of VMH^Ano4^ neurons ameliorates hyperglycemia in a type 1 diabetic (T1D) model. Finally, we showed that VMH^Ano4^ neurons represent a unique orexigenic VMH population that transmits positive valence, while stimulation of neurons that do not express Ano4 (VMH^non-Ano4^) in the VMH suppress feeding and transmit negative valence.

The ATP-sensitive potassium channel (K_ATP_ channel) has been well-established as a common ionic mechanism that mediates the glucose sensing in various GE populations, including GE neurons in the VMH (2, 16, 34, 35). However, the ionic mechanisms for GI glucose sensing appear to vary depending on the brain regions (36). For example, closure of leak potassium channels is shown to mediate hypoglycemia-induced activation of GLUT2-expressing neurons in the nucleus of the solitary tract (37). Low glucose activates neurons in the lateral hypothalamus through increasing NMDA-mediated glutamatergic currents (38). The cystic fibrosis transmembrane conductance regulator, a chloride ion channel, is implicated to mediate glucose-induced inhibition of Agouti-related peptide neurons (39) and VMH neurons (40). Here, we provide evidence to identify Ano4 as a common ionic mechanism for VMH GI sensing. The role of Ano4 in VMH GI neurons is first supported by single-cell RNA-Seq data from both mice and macaques, showing that Ano4 is highly overlapping with other putative molecular markers for VMH GI subpopulations. Furthermore, slice electrophysiology recordings confirmed that Ano currents are not present in GE or NGS neurons in the VMH, but exclusively exist in GI neurons located in all VMH subdivisions. Consistently, while a glucose load induces abundant c-fos expression in the VMH, none of these glucose-activated VMH neurons are Ano4-positive neurons, further suggesting that Ano4 is selectively expressed by GI neurons in the VMH. In the present study, we have demonstrated that 77.8% of VMH^Ano4^ neurons (as labeled by tdTomato in Ano4-P2A-Cre/Rosa26-LSL-tdTomato mice) are GI neurons, in which Ano current is present, and the other 22.2% of tdTomato^+^ neurons are not responsive to glucose. Importantly, none of these VMH^Ano4^ neurons are GE neurons. Two possibilities may account for the lack of Ano currents and GI properties in 22.2% tdTomato^+^ neurons. One possibility is that these neurons were labelled by tdTomato because Ano4 was transiently expressed earlier but no longer existed at the time of the experiment; in other words, these were Ano4 lineage neurons, but not current Ano4 neurons. The second possibility is that Ano4 was present in these neurons but it did not function as a membrane chloride channel to influence the excitability of the neuron. Of note, Ano4 has been implicated to function as a phospholipid scramblase (41). Nevertheless, we showed that genetic disruption of *Ano4* in VMH^SF1^ neurons largely reduced the percentage of GI neurons in the VMH. Together, these data indicate that Ano4 is a good maker for GI neurons in the VMH, although it may not be an exclusive GI marker.

Importantly, both pharmacological inhibition of Ano current and genetic disruption of Ano4 blunt low glucose-induced activation of VMH GI neurons. Thus, Ano4 is not only a GI marker, but is functionally required to mediate GI sensing. We further demonstrated that low glucose dose-dependently increased Ano currents in GI VMH^Ano4^ neurons. Consistent with the known role of the AMPK pathway in mediating VMH GI sensing (29), we showed that the AMPK blocker can abolish effects of low glucose to increase Ano currents and therefore prevent activation of GI VMH^Ano4^ neurons. We further demonstrated that genetic disruption of Ano4 in VMH neurons impaired 2-DG-induced glucose elevations and release of counterregulatory hormones (i.e., glucagon and corticosterone) during a hyperinsulinemic-hypoglycemic clamp. Further, activation of VMH^Ano4^ neurons promoted feeding and increased blood glucose associated with increases in corticosterone and norepinephrine. Unlike photoactivation of VMH^SF1^ neurons, which likely contain more VMH subpopulations (20), pancreatic hormones (e.g., insulin and glucagon) were not involved in activation of VMH^Ano4^ neurons. Notably, the hormonal responses following VMH^Ano4^ neuron activation were similar to those upon activation of Cckbr neurons in the VMH (26). Thus, our results support a model that hypoglycemia activates VMH GI neurons via the opening of Ano4 channel, and activation of these VMH GI neurons triggers release of counterregulatory hormones (e.g., corticosterone and norepinephrine) and promotes feeding to prevent severe hypoglycemia.

We identified the Ano4 channel as a relevant target for diabetic management. People with T1D receiving intensive insulin therapy are under a great risk of hypoglycemia, which could be life-threatening (42). Moreover, recurrent hypoglycemia impairs the counterregulatory responses and produces HAAF in people with T1D (42). Recent evidence indicates that inhibition of VMH subpopulation neurons (e.g., GcK, SF1, NOS1, and CCKBR) blunts counterregulatory response to hypoglycemia (20, 24–26). On the other hand, activation of these specific subpopulations mimics counterregulatory response to increase blood glucose. Interestingly, Flak and colleagues reported that silencing of VMH^CCKBR^ neurons significantly attenuates hyperglycemia in mice rendered diabetic by STZ treatment (26). In the present study, we found that genetic disruption of *Ano4* in the VMH blunted counterregulatory responses to hypoglycemia in a hyperinsulinemic-hypoglycemic clamp study as well as in 2-DG-induced glucopenia. Furthermore, impaired Ano4 functions in the VMH also contributed to the development of HAAF. More importantly, chronic inhibition of VMH^Ano4^ neurons significantly ameliorated STZ-induced diabetic hyperglycemia. Thus, impaired functions of VMH^Ano4^ neurons were implicated in dysregulated counterregulatory response to hypoglycemia, and hyperactivity of these same neurons may also contribute to diabetic hyperglycemia.

Notably, a recent large-scale human whole-exome sequencing study revealed that a mutation of the *ANO4* gene is associated with a higher BMI, although it is unclear whether this mutation causes gain-of-function or loss-of-function (43). In the present study, we found that female mice lacking Ano4 in the VMH show a modest body weight loss, while the same mutation did not affect body weight of male mice. Thus, we suggest that a higher BMI is associated with the gain-of-function of Ano4, and this association may differ in men and women. More detailed molecular characterizations of this BMI-associated ANO4 mutation, as well as the human genetic analysis, are needed to further delineate how the ANO4 mutation affects human body weight balance. Intriguingly, Stanley, et al. has reported that activation of VMH^GcK^ neurons promotes feeding and increases blood glucose (24). Of note, the mutation of the *GCK* gene has been associated with maturity-onset diabetes of young and persistent hyperinsulinemic hypoglycemia of infancy, but not with obesity (44, 45). Given the similar appetite-promoting and glucose-elevating effects by VMH^Ano4^ neurons, we suggest that the human ANO4 mutation may also be associated with dysregulations of glucose balance, a possibility that warrants further investigation.

Another interesting finding is that activation of VMH^Ano4^ neurons promotes feeding. The orexigenic nature of these VMH^Ano4^ neurons is consistent with functions of GI neurons that increase food intake to prevent severe hypoglycemia. This is somewhat surprising, considering that activation of the whole VMH population (labelled by SF1) inhibits food intake (21, 31, 32). We, therefore, suggest that VMH^Ano4^ neurons represent a unique orexigenic VMH subpopulation, while the rest of VMH neurons are largely anorexigenic. In line with this, we show that optogenetic stimulation of VMH^non–Ano4^ neurons (using a Cre-off approach) remarkably inhibits food intake, which is comparable to experimental stimulation of VMH^SF1^ neurons (20, 32). Further supporting the functional segregation of VMH^Ano4^ neurons versus other VMH neurons, we observed a positive valence triggered by VMH^Ano4^ neurons, while VMH^non–Ano4^ neurons transmit an opposing negative valence. The latter response is in line with defensive/avoidance, freezing, jumping, and escaping behaviors triggered by activation of subpopulations of VMH neurons, i.e., VMH^SF1^ and VMH^NOS1^ neurons (25, 31). Mechanisms underlying the opposite functions of these VMH subpopulations may include distinct projections and neurotransmitter/neuropeptide signals, which require further investigation to delineate (17).

The VMH is of critical importance for glucose homeostasis and feeding behavior. Here, we identified Ano4-mediated currents as a common mechanism for VMH GI sensing, and the Ano4 ion channel was physiologically required to trigger release of counterregulatory hormones and to promote feeding, which, in turn, prevented severe hypoglycemia. Further, VMH^Ano4^ neurons represented a unique orexigenic VMH subpopulation, surrounded by other anorexigenic neurons in the VMH. Considering the association of the ANO4 genetic mutation with abnormal BMI in humans, we suggest that the Ano4 ion channel and VMH^Ano4^ neurons are potential therapeutic targets for human diseases with abnormal feeding behavior and glucose imbalance.

## Methods

### Mice.

Several mouse strains were used for all experiments. SF1-Cre mice (Jackson Laboratory, 012462) and Rosa26-LSL-tdTomato allele mice (Jackson Laboratory, 007905) were obtained from the Jackson Laboratory. To examine the role of Ano4 in the VMH in energy metabolism, we generated Ano4 KO^SF1^ and their controls via CRISRP-Cas9 deletion. CRISPR-Cas9 deletion was used to generate mice lacking *Ano4* in VMH^SF1^ neurons, as described previously (27). In brief, SF1-Cre mice (male and female, 8–12 weeks of age) received stereotaxic injections of AAV-Ano4/sgRNAs-FLEX-tdTomato and AAV-FLEX-saCas9 into both sides of the VMH to selectively disrupt expression of Ano4 in VMH^SF1^ neurons. We included 2 control groups: WT mouse littermates that received the same viruses, and SF1-Cre mice that received AAV-FLEX-GFP and AAV-Ano4/sgRNAs-FLEX-tdTomato. Another cohort of male SF1-Cre mice with 1 side of the VMH injected with AAV-Ano4/sgRNAs-FLEX-tdTomato and AAV-FLEX-saCas9 (referred to as the KO side), and the other side of the VMH injected with AAV-FLEX-GFP and AAV-Ano4/sgRNAs-FLEX-tdTomato (referred to as the control side) were prepared for electrophysiological recordings of VMH^SF1^ control neurons and Ano4 KO VMH^SF1^ neurons, as well as validation of genomic deletion of Ano4 in VMH^SF1^ neurons.

To generate an Ano4-P2A-Cre knock-in mouse line, we used CRISPR-Cas9 gene editing to generate the in-frame genomic insertion of a P2A-Cre sequence 3′ to the final amino acid codon before the stop codon of Ano4. The self-cleaving P2A sequence allows for bicistronic expression of both endogenous Ano4 protein and Cre recombinase following translation of a single mRNA. The gene targeting was designed and performed by the Genetically Engineered Rodent Models Core at Baylor College of Medicine (BCM). Briefly, the sgRNA (5′-GAAAAGCACATCACAATGAG-3′) and the long single-stranded donor DNA (1,998 bp containing the P2A-Cre sequence flanked on the 5′ and 3′ sides with a 450 bp homology arm) were synthesized by Integrated DNA Technologies (IDT). The BCM Core microinjected Cas9 mRNA (100 ng/μl), ssDNA (100 ng/μl), and sgRNA (20 ng/μl) into the cytoplasm of 200 pronuclear stage C57Bl6j embryos, as previously described (46). Founder animals (F0) were screened for the correct insertion of the P2A-Cre sequence by PCR amplification of tail DNA using 2 primer pairs. The 5′ homology arm primers were: 5′-TGCAAACACTTAGCAATCTACACAG-3′ and 5′-GTACGGTCAGTAAATTGGACATAGG-3′. The 3′ homology arm primers were: 5′- TGAACTATATCCGTAACCTGGATAG-3′ and 5′-ATATAAGGCTTCACTCTATCTGACG-3′.

All the breeders have been backcrossed to C57Bl6j background for more than 12 generations. In addition, some C57Bl6j mice were purchased from the mouse facility of Baylor College of Medicine. To visualize Ano4-label neurons in the brain, we generated Ano4-P2A-Cre/Rosa26-LSL-tdTomato mouse line by crossing Ano4-P2A-Cre mouse line with Rosa26-LSL-tdTomato reporter mouse line. Ano4 neurons from these mice were therefore labeled by tdTomato.

Mice were housed in a temperature-controlled environment at 22–24°C, using a 12-hour light, 12-hour dark cycle. The mice were fed with regular chow (6.5% fat, 2920, HarlanTeklad). Water was provided ad libitum.

### Bioinformatic analysis of published data.

Analyses were performed in R 4.2.0 within RStudio 2022.02.2 Build 485 utilizing Seurat 4.0.4. Count matrix, features, barcodes, and metadata were downloaded from https://www.ncbi.nlm.nih.gov/geo/query/acc.cgi?acc=GSE172204 and https://www.ncbi.nlm.nih.gov/geo/query/acc.cgi?acc=GSE172203 and were loaded into Seurat objects. Data were processed in Seurat using the same parameters published in Affinati et al. (28), with the aid of custom code retrieved from https://github.com/alanrupp/affinati-elife-2021/blob/master/experiments/snRNA-seq/analysis/mouse/mouse.Rmd Seurat objects were filtered for cells labeled VMH_cluster using metadata. Count data were scaled and functions “RunPCA” and “RunUMAP” were executed. Cells in VMH clusters were plotted in uniform manifold approximation and projection (UMAP) space using function “DimPlot”. Count data were normalized using “SCTransform” and expression of select genes were shown using “FeaturePlot” function. The function “DotPlot” was used to show the percentage of cells within each cluster that expressed select genes as well as the average expression of select genes for each cluster. Heatmaps were constructed manually by filtering for cells expressing combinations of select genes and counting those cells using functions from the package dplyr 1.0.9. We then calculated percentages and color coded the percentages, rounding to the nearest 10%.

### Electrophysiology.

Male WT C57Bl6j, SF1-Cre, and Ano4-P2A-Cre/Rosa26-LSL-tdTomato mice were used for electrophysiological recordings. Mice were deeply anesthetized with isoflurane and transcardially perfused with a modified ice-cold sucrose-based cutting solution (pH 7.3) containing 10 mM NaCl, 25 mM NaHCO_3_, 195 mM sucrose, 5 mM glucose, 2.5 mM KCl, 1.25 mM NaH2PO_4_, 2 mM Na-pyruvate, 0.5 Mm CaCl_2_, and 7 mM MgCl_2_ (all chemicals listed here were purchased from Sigma-Aldrich), bubbled continuously with 95% O_2_ and 5% CO_2_ (47). The mice were then decapitated, and the entire brain was removed and immediately submerged in the cutting solution. Slices (250 μm) were cut with a Leica VT1000 S vibrating microtome (Leica Biosystems). Then, 3 to 4 brain slices containing the VMH were obtained for each animal (bregma −2.06 mm to −1.46 mm; interaural 1.74–2.34 mm). The slices were recovered for 1 hour at 34°C and then maintained at room temperature in artificial cerebrospinal fluid (aCSF, pH 7.3) containing 126 mM NaCl, 2.5 mM KCl, 2.4 mM CaCl_2_, 1.2 mM NaH_2_PO_4_, 1.2 mM MgCl_2_, 5.0 mM glucose, and 21.4 mM NaHCO_3_ saturated with 95% O_2_ and 5% CO_2_ before recording.

Slices were transferred to a recording chamber and allowed to equilibrate for at least 10 minutes before recording. The slices were superfused at 34°C in oxygenated aCSF at a flow rate of 1.8–2 mL/min. Patch pipettes with resistances of 3–5 MΩ were filled with intracellular solution (pH 7.3) containing 128 mM K-gluconate, 10 mM KCl, 10 mM HEPES, 0.1 mM EGTA, 2 mM MgCl_2_, 0.05 mM Na-GTP, and 0.05 mM Mg-ATP. Recordings were made using a MultiClamp 700B amplifier (Axon Instrument), sampled using Digidata 1440 A and analyzed offline with pClamp 10.3 software (Axon Instruments). Series resistance was monitored during the recording, and the values were generally under 10 MΩ and were not compensated. The liquid junction potential was +12.5 mV and was corrected after the experiment. Data were excluded if the series resistance increased dramatically during the experiment or were without overshoot for action potential. Currents were amplified, filtered at 1 kHz, and digitized at 10 kHz.

Neurons in the dmVMH, cVMH, and vlVMH were randomly recorded in brain slices prepared from 10–12 week-old male C57Bl6j mice. In some experiments, tdTomato-labeled neurons in the VMH from male Ano4-P2A-Cre/Rosa26-LSL-tdTomato mice were recorded under different concentrations. In another experiment, tdTomato^+^ SF1 neurons (Ano4 KO^SF1^) and GFP positive SF-1 neurons (control) were used for the validation of Ano4 in VMH SF1 neurons. Current-clamp was engaged to test neural firing frequency and resting membrane potential at the baseline of 5 mM glucose aCSF and 1 mM glucose aCSF. The values for resting membrane potential and firing frequency were averaged within a 2-minute bin at the 5 mM glucose or 1 mM glucose aCSF condition. The lucifer yellow (Sigma-Aldrich) dissolved in pipette solution (1 μM) was used to validate the location of recorded VMH neurons from male C57Bl6j mice. TdTomato-labeled neurons in the VMH were visualized using epifluorescence and IR-DIC imaging on an upright microscope (Eclipse FN-1, Nikon) equipped with a movable stage (MP-285, Sutter Instrument). We define GI, GE, or nonglucose sensing (NGS) neurons by calculating the resting membrane potential changes caused by 1 mM glucose treatment. If the resting membrane potential of a neuron was depolarized at least 2mV in amplitude by 1 mM glucose aCSF, we defined it as a GI neuron. If the resting membrane potential of a neuron was hyperpolarized at least 2mV in amplitude by 1 mM glucose aCSF, we defined it as a GE neuron. If the resting membrane potential of a neuron was changed less than 2mV in amplitude by 1 mM glucose aCSF, we defined it as a NGS neuron. After the identification of each neuron, the same neuron was recorded under 5 mM and 1 mM glucose aCSF in the presence of an Ano blocker, 100 μM CaCCinh-A01 (48).

To measure Ano currents, the pipette solution contained (in mM): CsCl 130, NaH_2_PO_4_ 1.2, Na_2_HPO_4_ 4.8, EGTA, MgCl_2_ 1.0, D-glucose 5.0, and ATP 3.0 (pH adjusted to 7.2). The total Ano current was recorded under voltage-clamp by holding the membrane potential at −20 mV in 5 mM glucose or 1 mM glucose aCSF in the presence of 1 μM Tetrodotoxin (TTX), 100 μM 4-Aminopyridine (4-AP), and 100 μM Tetraethylammonium chloride (TEA-Cl). At intervals, neurons were voltage-clamped from −50 mV to 50 mV in steps of 10 mV for 1 second (49). The neurons were treated with the Ano blocker, 100 μM CaCCinh-A01, for 3 minutes. The Ano current was calculated by subtracting the left current in the presence of CaCCinh-A01 from total current without the blocker.

To test if low glucose treatment directly regulated the neuronal activity of VMH glucose-sensing neurons, VMH neurons were pretreated with a cocktail of synaptic blockers containing 1 μM TTX (a reversible, selective, and high-affinity inhibitor of voltage-gated sodium channels), 30 μM CNQX (a potent non-NMDA glutamate receptor antagonist), 30 μM D-AP5 (a potent and selective NMDA receptor antagonist), and 50 μM bicuculline (a GABA_A_ receptor antagonist) to block the excitatory and inhibitory synaptic inputs in the recorded VMH neurons. Resting membrane potential was calculated after 1 mM glucose aCSF treatment with or without 100 μM of the Ano blocker, CaCCinh-A01. To test whether low glucose-activated VMH^Ano4^ neurons can be blocked by AMPK blocker, identified GI VMH^Ano4^ neurons were pretreated with the AMPK blocker compound C (10 μM). Ano currents, resting membrane potential, and firing frequency were measured as described above.

In some recordings, fluorescent-guided whole-cell patch-clamp recordings were performed in Kir2.1-dTomato-expressing VMH neurons in Ano4-P2A-Cre mice. The baseline of neuronal firing frequency and resting membrane potential was compared in VMH neurons expressing Kir2.1-dTomato.

### RNAscope.

Male Ano4-P2A-Cre/Rosa26-LSL-tdTomato mice (8–12 weeks of age) were anesthetized and perfused with 0.9% saline followed by 10% formalin. Brains were removed and post fixed in 10% formalin for 16 hours at 4°C and cryoprotected in 30% sucrose for 48 hours. Brains were frozen and sectioned at 14 μm using the cryostat and washed in DEPC-treated PBS for 10 minutes. Sections were mounted on charged slides, dried for 0.5 hours at room temperature and stored at −80°C. On the day of the RNAscope assay, the slides were thawed and rinsed 2 times in PBS and baked in an oven for 30 minutes at 60 °C. After that, slides were post fixed in 10% formalin for 15 minutes at 4 °C. Slides were then gradually dehydrated in ethanol (50%, 70% and 100%, 5 minutes each) and underwent target retrieval for 5 minutes at 100°C. After being incubated in protease III (Advanced Cell Diagnostics) for 30 minutes at 40°C, slides were rinsed in distilled water and incubated in mouse RNAscope probes for *Ano4* (439551-C1, Advanced Cell Diagnostics) and *tdTomato* (317041-C3, Advanced Cell Diagnostics) for 2 hours at 40°C. Sections were then processed using RNAscope Fluorescent Multiplex Detection Reagents (320851, Advanced Cell Diagnostics) according to the manufacturer’s instructions. Slides were cover-slipped and analyzed using an Andor BC43 Benchtop Confocal (Oxford Instruments).

### IHC.

Six male Ano4-P2A-Cre/Rosa26-LSL-tdTomato mice (8–12 weeks of age) were used to characterize saline (10 mL/kg, i.p.) or glucose (2 g/kg, i.p.)–induced c-fos in the VMH. Mice were fasted for 2 hours before saline or glucose treatment. Ninety minutes after injection, mice were anesthetized with inhaled isoflurane and quickly perfused with saline followed by 10% formalin. After dehydration with 30% sucrose, the brains were cut into sections of 25 μm. Sections from each mouse were blocked with 3% normal donkey serum for 2 hours and incubated with rabbit anti-c-Fos antibody (1:1,000, 226003, Synaptic Systems) on a shaker at 4°C overnight, followed by the donkey anti-rabbit Alexa Fluor 488 (1:500, A21206, Invitrogen) for 2 hours at room temperature. Slides were cover-slipped and analyzed using a fluorescence microscope. The numbers of c-fos and c-fos/Ano4 double-positive cells in the VMH were counted. Three mice were included in each group for statistical analyses.

### Food intake, body weight, and energy expenditure.

SF1-Cre and their littermate WT mice were singly housed 1 week before stereotaxic surgery. SF1-Cre mice received stereotaxic injections of AAV-Ano4/sgRNAs-FLEX-tdTomato and AAV-FLEX-saCas9 into both sides of the VMH to disrupt expression of Ano4 selectively in VMH^SF1^ neurons. WT mouse littermates received the same viruses, and SF1-Cre mice received with AAV-FLEX-GFP and AAV-Ano4/sgRNAs-FLEX-tdTomato were combined as control group. These mice were provided with regular chow until the end of the study. Food intake, body weight, and basal blood glucose were measured every week after surgery. On the day of glucose measurement, mice were brought to the procedure room for 2 hours in the morning, during which food was removed to ensure that they had an empty stomach. Glucose was then measured. Quantitative magnetic resonance was used to determine body composition. Energy expenditure measurements were performed in temperature-controlled (23°C) cabinets containing 16 TSE PhenoMaster metabolic cages. Mice were acclimatized to the metabolic cages for 3 days. Data collected from days 3 and 4 were used for analyses and energy expenditure was analyzed using the online CalR tool (50, 51).

### GTT, ITT, and 2-DG assay.

For GTT, after an overnight fast, mice received i.p. injections of 1.5 g/kg D-glucose (G8270, Sigma-Aldrich) in the morning. Blood glucose was measured from tail blood using a glucometer (OneTouch Ultra) at (0, 15, 30, 60, and 120 minutes. For ITT, after a 2-hour fast in the morning, mice received i.p. injections of insulin (1.5 U/kg). Blood glucose was measured at 0, 15, 30, 60, and 120 minutes. For the 2-DG assay, mice were fasted for 2 hours in the morning, followed by 2-DG (300 mg/kg, i.p.) treatment. Blood glucose was then measured at 0, 15, 30, 60, and 120 minutes after injections. For the HAAF paradigm, mice were treated with 2-DG (300 mg/kg, i.p.) for 4 consecutive days in the morning after 2-hour fasts and glucose levels were measured on day 1 and day 4, as indicated.

### Hyperinsulinemic-hypoglycemic clamp.

Male Ano4 KO^SF1^ mice and their control mice were sent to the NIH-funded Baylor Mouse Metabolism & Phenotyping Core for the hyperinsulinemic-hypoglycemic clamp studies. As we have previously described (52), a microcatheter was inserted into the jugular vein by survival surgery and mice were given 4 to 5 days for complete recovery. Studies were then performed in conscious mice. Overnight-fasted conscious mice were primed with regular insulin (bolus 10 mU/kg body weight) followed by an approximately 2-hour constant insulin infusion (10 mU/kg/min). Using a separate pump, 25% glucose was used to maintain the blood glucose level at 50 mg/dL, as determined every 6–9 minutes using a glucometer (LifeScan). The glucose infusion rate (GIR) was then recorded continuously and blood samples were collected at a 90 minute time point. Blood samples were collected during the hypoglycemic condition and processed to obtain plasma. Plasma glucagon and corticosterone levels were measured using the mouse glucagon (10-1281-01, Mercodia) and corticosterone (ADI-900-097, Enzo Life) ELISA kits according to the manufacturer’s instructions.

### Validation of Genomic Deletion of Ano4 in VMH^SF1^ Neurons.

Male SF1-Cre mice were prepared with 1 side of the VMH injected with AAV-Ano4/sgRNAs-FLEX-tdTomato and AAV-FLEX-saCas9 and the other side of the VMH injected with AAV-FLEX-GFP, and AAV-Ano4/sgRNAs-FLEX-tdTomato was used as a control. To detect if the CRISPR-Cas9 approach successfully induced the mutation of Ano4, 2-step touchdown PCR was performed with each reaction containing 1 single SF1 neuron that was handpicked under the microscope. The 1st primer pair across the sgRNA target region of Ano4, 5′-AGCGCAGCTCACCTTCTAAC-3′ and 5′-AATCTTGCTCTGCACACGCT-3′ (750 bp), were used for the first step of PCR. The PCR products were then used for the second step of PCR with the primer pair: 5′-GGGGCAGGCAGGTTTTACAT-3′ and 5′-CACACAGACCTATGACCCCC-3′ (450 bp). To ensure the success of the neuron picking, 2 control primer pairs for an irrelevant gene (i.e., *Gabra5*) were also included to amplify the nonrelevant region of the genome. The first primer pair was: 5′-CCTGTAAGAGTAGCCTGGCAT-3′ and 5′-AGATAAGAGACGTGGGGCTG-3′ (744 bp), and the second primer pair was: 5′-AAGGAATCCAGTGACCAGCC-3′ and 5′-TCCTAAGGAACCAGCATGGG-3′ (525 bp) (53).

To measure the Ano4 protein expression in control SF1 neurons and Ano4 KO^SF1^ neurons, the VMH was dissected out as described here. After brief anesthetization with isoflurane, mice were decapitated and the whole brain was removed. Frontal sections of the hypothalamus were prepared using a brain matrix (1 mm thick), and the VMH was microdissected under a fluorescence stereomicroscope (Nikon, stereozoom SMZ1500), frozen immediately in dry ice, and stored at –80°C. For immunoblot assay, the tissue samples were lysed with RIPA buffer (Alfa Aesar) with a protease inhibitor cocktail and phosphatase inhibitors. The lysates were subsequently sonicated with 5 seconds pulse at 20% power using a probe sonicator and incubated on ice for 30 minutes. Lysates were centrifuged at 18000*g* for 15 minutes at 4°C, and supernatants containing protein extracts were subjected to SDS-PAGE and immunoblot assay. The proteins were electrophoresed on a 10% SDS- polyacrylamide gel and subsequently transferred to PVDF membrane. The membranes were probed with an anti-Ano4 antibody (1:200, #MBS8506049, Biocompare) and an anti-GAPDH antibody (1:1,000, 92310SF, Cell Signaling Technology). After incubation with primary antibodies overnight at 4°C, the membranes were then incubated with anti-rabbit IgG HRP-linked antibody (7074s, Cell Signaling Technology). For signal development, target bands were detected using darkroom development techniques for chemiluminescence. Bands were quantified using ImageJ software. See complete unedited blots in the supplemental material.

### Designer receptors exclusively activated by designer drugs.

Ano4-P2A-Cre and WT littermate male mice (8–10 weeks of age) were anesthetized by isoflurane and received stereotaxic injections of pAAV8/hSyn-DIO-hM3D(Gq)-mCherry (3.2 × 10^13^ VG/mL, #44361, Addgene) in the VMH (AP: + 1.70 mm, ML: + 0.43 mm, DV: +5.62 mm). All mice were allowed to recover for 4 weeks after surgery. Satiated or overnight-fasted mice received i.p. injections of clozapin N-oxide (CNO, 3 mg/kg; Cayman Chemical Inc.) in the morning, with food presented 15 minutes later. Food intake was measured at series of time points as indicated in the figures. For glucose measurement, mice were deprived from food from 2 hours before the CNO injection in the morning; glucose was measured at 15, 30, 60, and 120 minutes after i.p. injections of CNO during which period food was absent from the cages.

In separate studies, plasma insulin, glucagon, corticosterone, norepinephrine, and epinephrine levels following CNO injection were measured using ELISA kits for mouse insulin (90080, Crystal Chem Inc.), mouse glucagon (10-1281-01, Mercodia), corticosterone (ADI-900-097, Enzo Life), mouse norepinephrine (KA1891, ABNOVA), and mouse epinephrine (KA3837, ABNOVA) according to the manufacturer’s instructions.

### STZ treatment.

Ano4-P2A-Cre mice and WT littermate male mice (8–12 weeks of age) were anesthetized by isoflurane and received stereotaxic injections of AAV-EF1a-DIO-Kir2.1-P2A-dTomato (30). After a 4-week recovery, mice were treated with streptozotocin, (STZ, i.p., 160 mg/kg, Millipore Sigma) every week for 2 weeks. STZ was administered immediately after being dissolved into freshly prepared sodium citrate buffer (0.1 M, PH 4.5). Body weight and blood glucose were measured every other day. Plasma insulin, leptin, glucagon, and corticosterone levels were measured using ELISA kits for mouse insulin (90080, Crystal Chem Inc.), leptin (ADI-900-019A, Enzo Life), glucagon (10-1281-01, Mercodia), and corticosterone (ADI-900-097, Enzo Life) according to the manufacturer’s instructions.

### Optogenetic stimulation.

We stereotaxically injected AAV-EF1α-DIO-hChR2(H134R)-EYFP into the VMH (200 nL, 6.2x10^12^ GC per ml) of Ano4-P2A-Cre male mice (8–12 weeks of age) to express ChR2 specifically in VMH^Ano4^ neurons. To express ChR2 in the non-Ano4 neurons in the VMH, pAAV-Ef1a-DO-hChR2(H134R)-mCherry (4.1x10^12^ GC per mL, packaged by core facility of Baylor, 37082, Addgene) were injected into the VMH of male Ano4-P2A-Cre mice (8–12 weeks of age). Optogenetic fibers were placed 0.3–0.4 mm above the injection site. Mice were allowed at least 4 weeks for recovery before experimental stimulation. Blue light (473 nm, 10 ms/pulse, 20 Hz; MGL-FN-589, CNI LASER) was used to experimental stimulation, while yellow light (595 nm, 10 ms/pulse, 20 Hz; MGL-FN-589, CNI LASER) was used as the control. Feeding behavior and blood glucose levels were measured similarly as described above.

### Real-time place preference and open field test.

The same mice used in the aforementioned optogenetic studies were used here. All tests were performed in a dedicated sound-proof behavioral facility. These mice were brought to the procedure room 2 hours before the start of each test and remained in the same room throughout the test. The conditioned place preference apparatus contained 2 identical conditioning chambers (chamber 1 and 2) that were connected by an opening (12.5 cm) in the center. Each chamber was 50 × 50 × 25 cm (length × width × height) with black pexiglass wall and white pexiglass floor. Blue light (473 nm, 10 ms/pulse, 20 Hz) was shone whenever the mouse entered chamber 2 and ceased when it entered chamber 1. Yellow light (595 nm, 10 ms/pulse, 20 Hz) was used for the controls in a different trail. Each trial contained a 10-minute recording.

The open-field test was performed in a clear Plexiglas open-field arena (40 cm × 40 cm × 30 cm). Mice were placed into the center of the arena and allowed to explore for 5 minutes as baseline, followed by 5 minute blue (473 nm, 10 ms/pulse, 20 Hz) or yellow light (595 nm, 10 ms/pulse, 20 Hz) stimulation.

### Statistics.

The minimal sample size was predetermined by the nature of experiments and previous experience. For most of the physiological readouts (body weight, food intake, etc.), 5–10 mice per group were included. The data are presented as mean ± SEM unless otherwise stated. Statistical analyses were performed using GraphPad Prism 9.0 to evaluate normal distribution and variations within and among groups. One-way and 2-way ANOVAs followed by Bonferroni’s adjustment as well as 2-tailed, unpaired and paired Student’s *t* tests were used for statistical analyses. Methods of statistical analyses were chosen based on the design of each experiment and are indicated in figure legends. *P* < 0.05 was considered to be statistically significant.

### Study approval.

Care of all animals and procedures were approved by Baylor College of Medicine IACUC.

### Data availability.

Data are available from the corresponding author upon request.

## Supplementary Material

Supplemental data

## Version 1 06/01/2023

In-Press Preview

## Version 2 07/17/2023

Electronic publication
