# Supplementary material for: Anoctamin 4 channel currents activate glucose-inhibited neurons in the mouse ventromedial hypothalamus during hypoglycemia
Source: J Clin Invest. 2023 Jul 17;133(14):e163391. doi: 10.1172/JCI163391 (PMC10348766; doi:10.1172/JCI163391)
Supplement: Supplemental data [file jci-133-163391-s017.pdf]

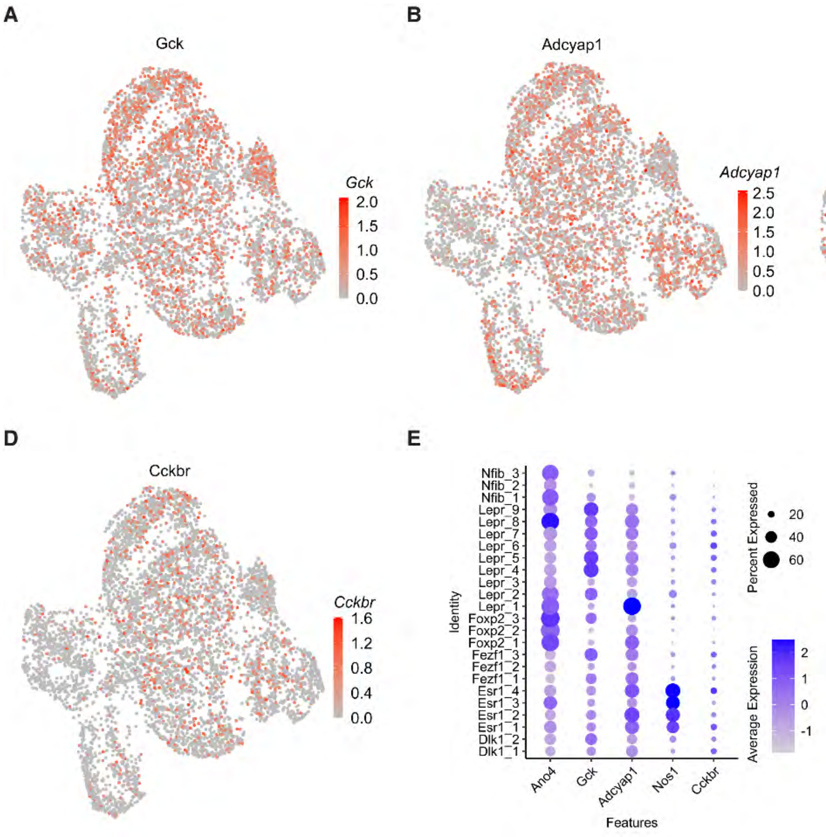
A
Gck
B
Adcyap1
C
Nos1
D
Cckbr
E
Percent Expressed
20
40
60
Average Expression
2
1
0
-1
Identity
Features
Ano4
Gck
Adcyap1
Nos1
Cckbr
Nfib_3
Nfib_2
Nfib_1
Lepr_9
Lepr_8
Lepr_7
Lepr_6
Lepr_5
Lepr_4
Lepr_3
Lepr_2
Lepr_1
Foxp2_3
Foxp2_2
Foxp2_1
Fezf1_3
Fezf1_2
Fezf1_1
Esr1_4
Esr1_3
Esr1_2
Esr1_1
Dlk1_2
Dlk1_1

Supplementary Figure 1. Distribution of putative markers of GI neurons in the VMH.

A-I. Gck, Adcyap1, Nos1 and Cckbr expression in VMH neurons mapped in same UMAP space. **J**. Dotplot of VMH neuron clusters for target genes. Size of dot indicating the percent of neurons in that cluster that express the target gene. Color of dot indicating the average expression of target gene in that cluster. Expression data presented normalized using Variance Stabilizing Transformations for Single Cell UMI Data (SCTransform). (Relative to **Figure 1**)

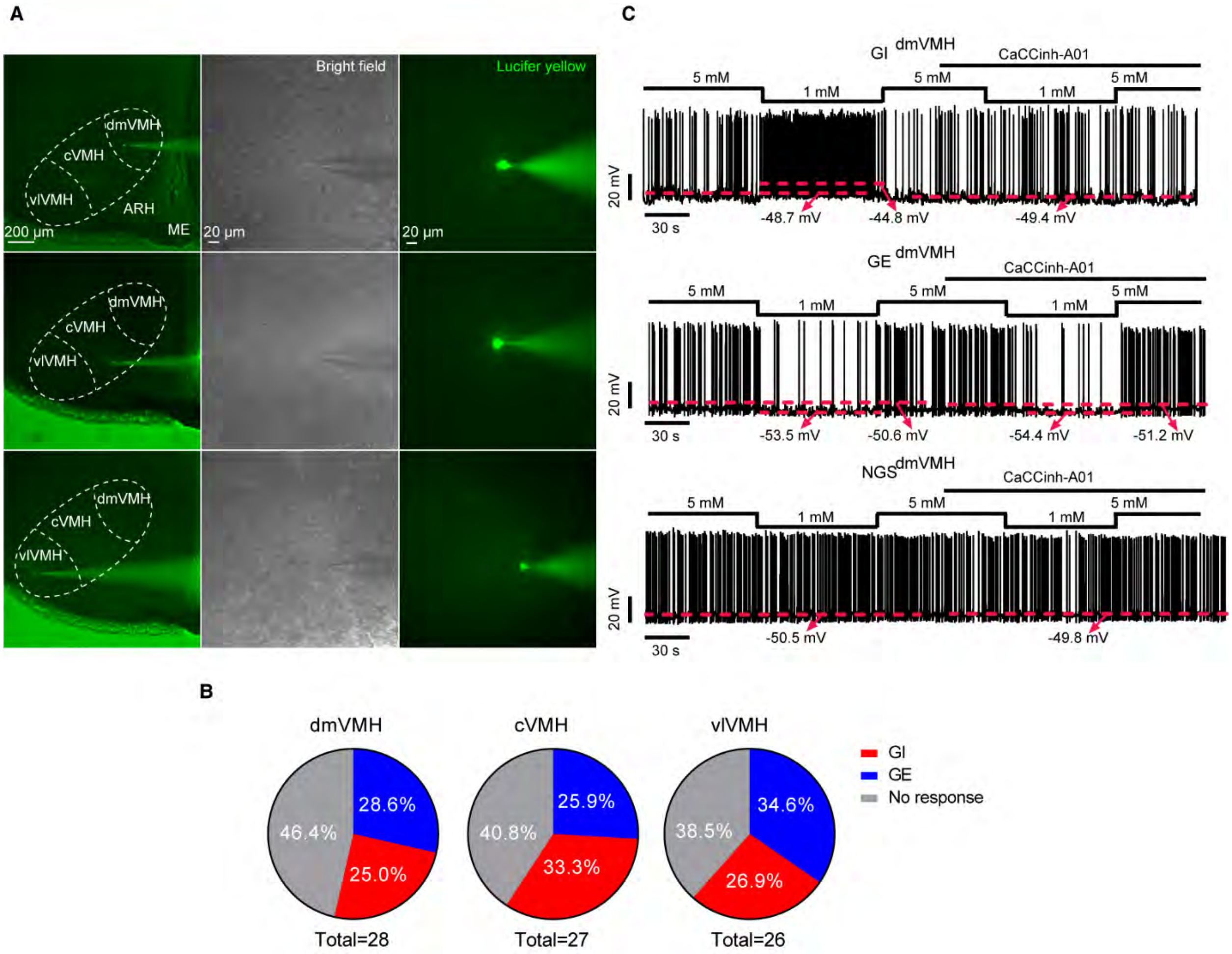
A
Bright field
Lucifer yellow
dmVMH
cVMH
vlVMH
ARH
ME
200 μm
20 μm
20 μm
C
GI dmVMH
CaCCinh-A01
5 mM
1 mM
5 mM
1 mM
5 mM
20 mV
30 s
-48.7 mV
-44.8 mV
-49.4 mV
GE dmVMH
-53.5 mV
-50.6 mV
-54.4 mV
-51.2 mV
NGS dmVMH
-50.5 mV
-49.8 mV
B
dmVMH
cVMH
vlVMH
GI
GE
No response
28.6%
46.4%
25.0%
25.9%
40.8%
33.3%
34.6%
38.5%
26.9%
Total=28
Total=27
Total=26

906

Supplementary Figure 2. Electrical responses of VMH neurons to glucose fluctuation.

A. Experimental illustration of neurons recorded randomly in the dmVMH, cVMH and vlVMH.

B. Percentage of GI, GE and NGS neurons in different VMH subdivisions (n=28 for dmVMH, n=27 for cVMH, and n=26 for vlVMH).

C. Representative electrophysiological responses to glucose fluctuation (5 → 1 mM) in a GI, GE and NGS neuron in the absence or the presence of the Ano inhibitor CaCCinh-A01. (Relative to **Figure 1**)

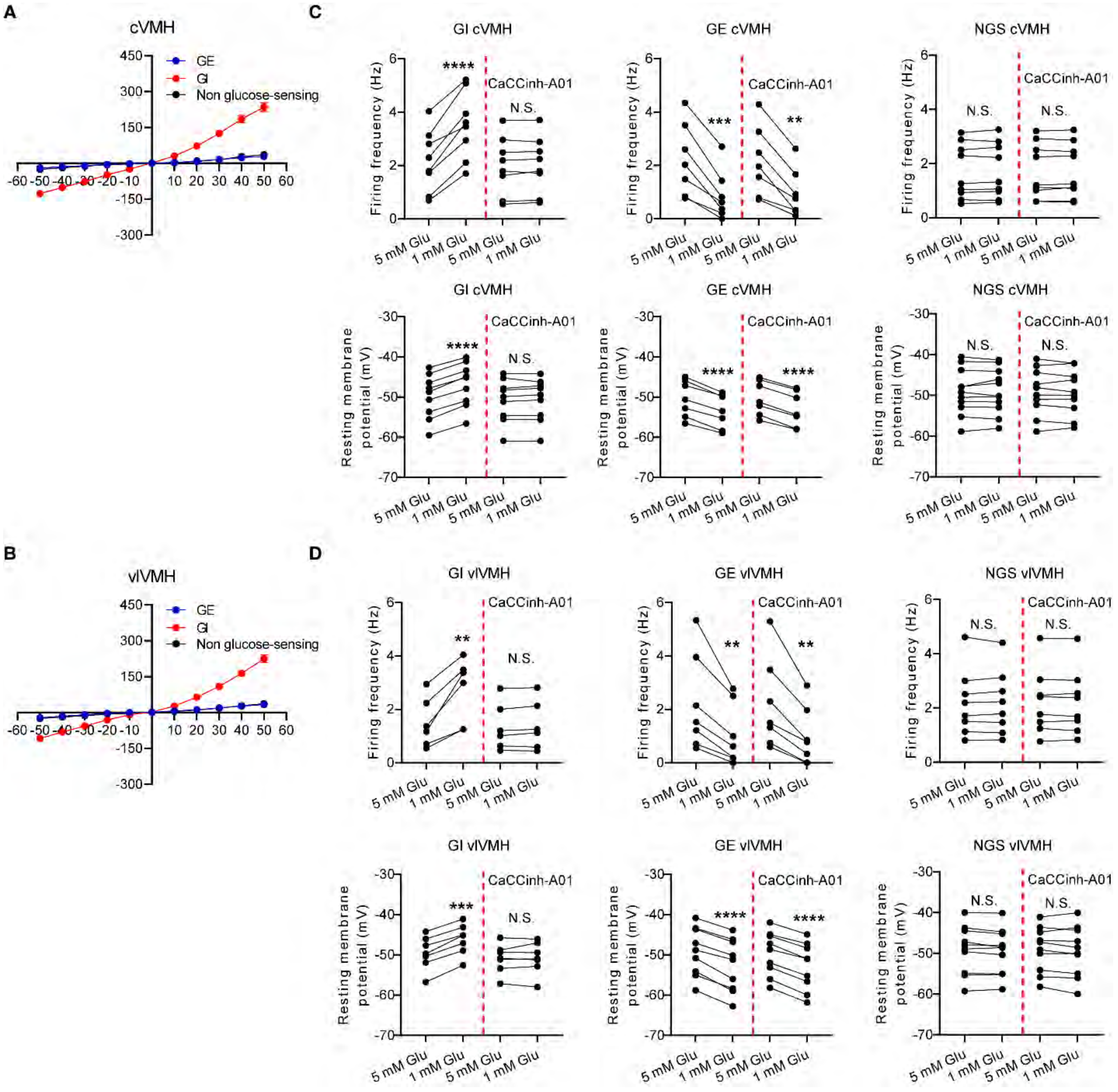
A
cVMH
GE
GI
Non glucose-sensing
B
vlVMH
C
GI cVMH
GE cVMH
NGS cVMH
Firing frequency (Hz)
Resting membrane potential (mV)
CaCCinh-A01
N.S.
5 mM Glu
1 mM Glu
D
GI vlVMH
GE vlVMH
NGS vlVMH

915

Supplementary Figure 3. Ano4 mediates glucose sensing of cVMH and vlVMH GI neurons

A-B. Ano current was detected in GI neurons, but not in GE or NGS neurons in the cVMH (n=5 for GE, n=5 for GI, and n=8 for NGS) and vlVMH (n=7 for GE, n=7 for GI, and n=8 for NGS).

C-D. Firing frequency and resting membrane potential of GI, GE and NGS neurons in the cVMH and vlVMH under glucose exposure from 5 → 1 mM in the absence or the presence of an Ano inhibitor CaCCinh-A01 (n=7 for GE, n=7 for GI, and n=8 for NGS in cVMH, and n=7 for GE, n=6 for GI, and n=8 for NGS in vlVMH). Data are expressed as mean ± standard errors of the mean. Significant differences between 5 mM glucose and 1 mM glucose are shown as $^{**}p < 0.01$, $^{***}p < 0.001$ and $^{****}p < 0.0001$ (Paired t-test for **C and D**). N.S. represents non-significant. (Relative to **Figure 1**)

929

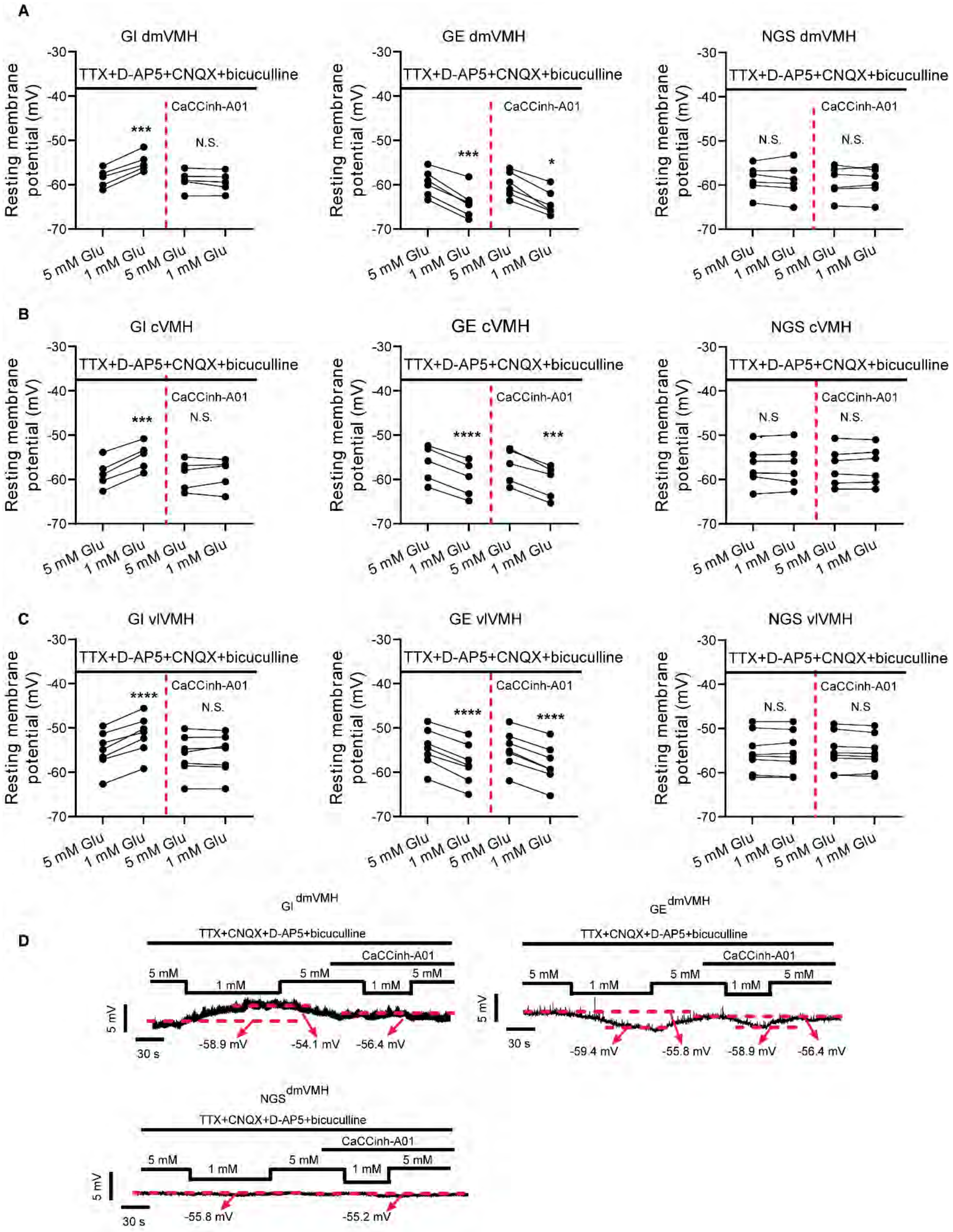
A
GI dmVMH
GE dmVMH
NGS dmVMH
TTX+D-AP5+CNQX+bicuculline
CaCCinh-A01
Resting membrane potential (mV)
5 mM Glu
1 mM Glu
***
N.S.
*
B
GI cVMH
GE cVMH
NGS cVMH
****
N.S
C
GI vlVMH
GE vlVMH
NGS vlVMH
D
GI dmVMH
GE dmVMH
NGS dmVMH
TTX+CNQX+D-AP5+bicuculline
5 mM
1 mM
5 mV
30 s
-58.9 mV
-54.1 mV
-56.4 mV
-59.4 mV
-55.8 mV
-58.9 mV
-56.4 mV
-55.8 mV
-55.2 mV

Supplementary Figure 4. Electrical responses of VMH neurons to glucose fluctuation in the presence of a cocktail of synaptic blockers.

A-C. Effect of the Ano inhibitor CaCCinh-A01 on membrane potential of GI, GE and NGS neurons in responses to glucose fluctuation in the presence of a cocktail of synaptic blockers (TTX, CNQX, D-AP5 and bicuculline) (n=5-6 for dmVMH, n=5-6 for cVMH, and n=7-8 for vlVMH).

D. Representative membrane potential of a GI, GE and NGS neuron in dmVMH in response to glucose fluctuations (5 → 1 mM) in the presence of a cocktail of synaptic blockers (TTX, CNQX, D-AP5 and bicuculline).

Data are expressed as mean ± standard errors of the mean. Significant differences between 5 mM glucose and 1 mM glucose are shown as *$p < 0.05$, ***$p < 0.001$ and ****$p < 0.0001$ (paired t-test). N.S. represents non-significant. (Relative to **Figure 1**)

943

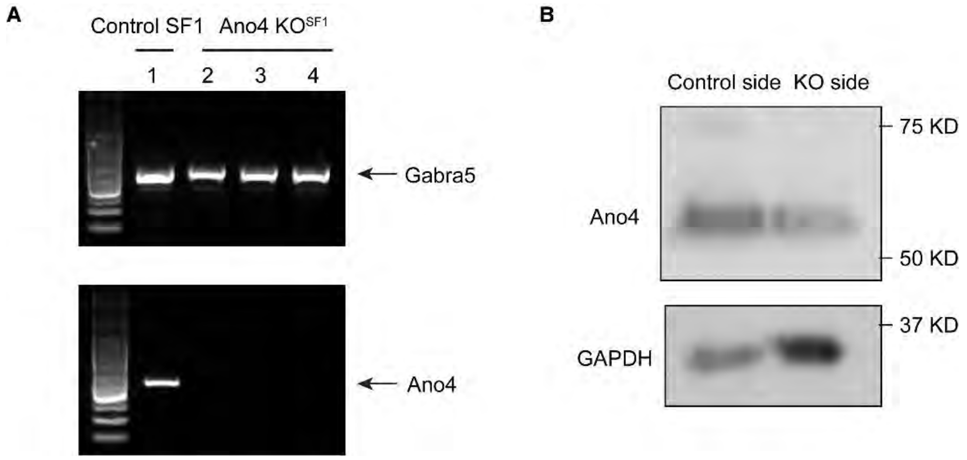

A
Control SF1
Ano4 KO^SF1
1
2
3
4
Gabra5
Ano4
B
Control side
KO side
75 KD
Ano4
50 KD
37 KD
GAPDH

Supplementary Figure 5. Validation of Ano4 deletion in VMH^{SF1} neurons.

A. Representative image for the single neuron PCR to detect potential deletion of Ano4. TdTomato-labeled single SF-1 neuron (target) and non-tdTomato-labeled control neuron (non-target) were picked under the microscope. The CRISPR targeted *Ano4* locus was PCR amplified and PCR products were resolved by agarose gel electrophoresis. The irrelevant gene (*Gabra5*) was also amplified as a control for the integrity of genome samples.

B. Ano4 protein level in control side of VMH and KO side of VMH. (Relative to **Figure 2**)

952

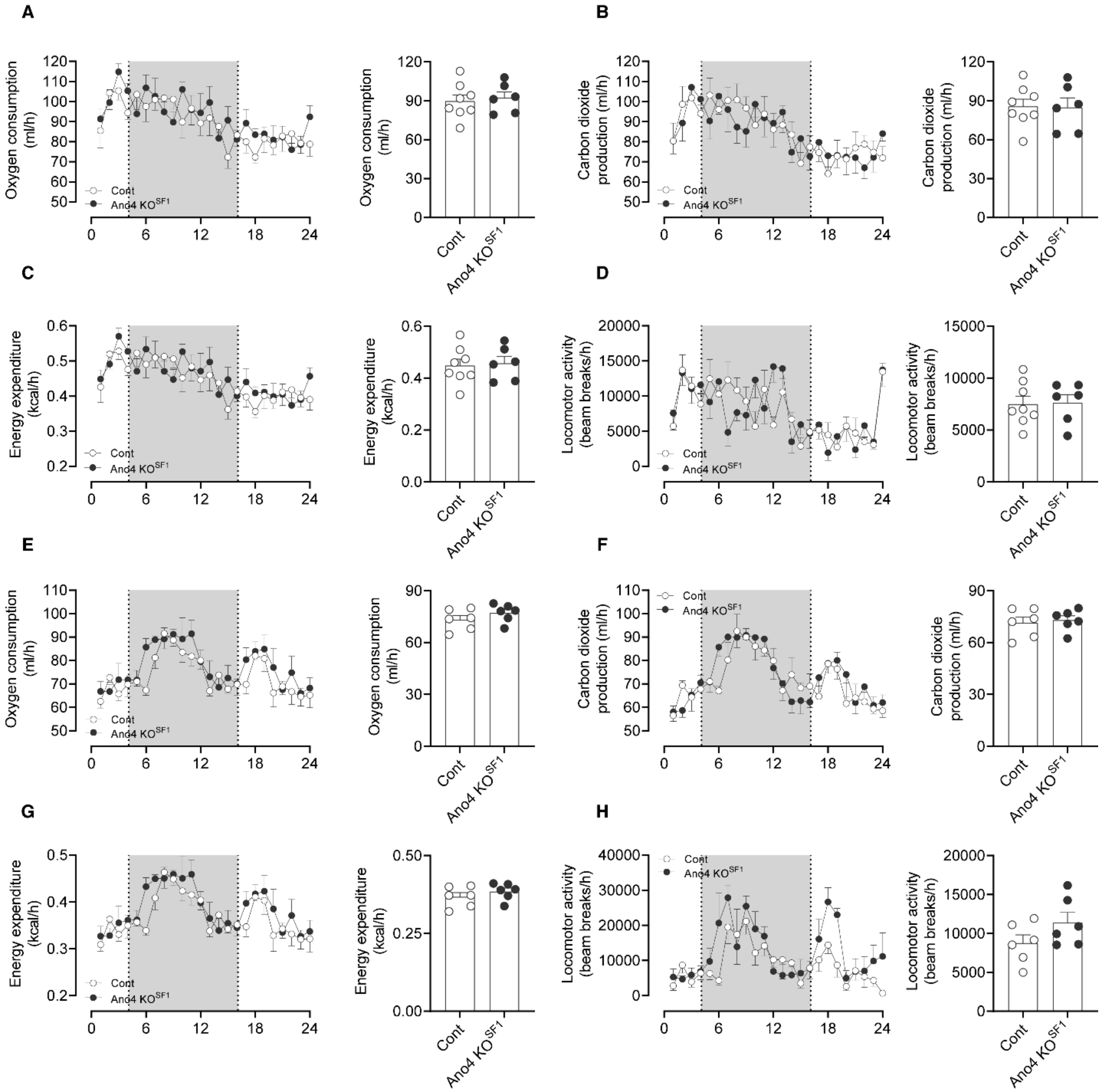
A
B
C
D
E
F
G
H
Oxygen consumption (ml/h)
Carbon dioxide production (ml/h)
Energy expenditure (kcal/h)
Locomotor activity (beam breaks/h)
Cont
Ano4 KO^{SF1}

Supplementary Figure 6. Knockout of *Ano4* in the VMH^{SF1} neurons did not alter energy expenditure in both male and female mice.

A-**D**. Oxygen consumption, carbon dioxide production, energy expenditure and locomotor activity in male mice (n=8 for Cont, and n=6 for Ano4 KO^{SF1}).

E-**H**. Oxygen consumption, carbon dioxide production, energy expenditure and locomotor activity in female mice (n=6 for Cont and Ano4 KO^{SF1}).

Data are expressed as mean ± standard errors of the mean. There was no significant difference between Cont (Control) vs. Ano4 KO^{SF1} mice for all collected parameters. (Relative to **Figure 2**)

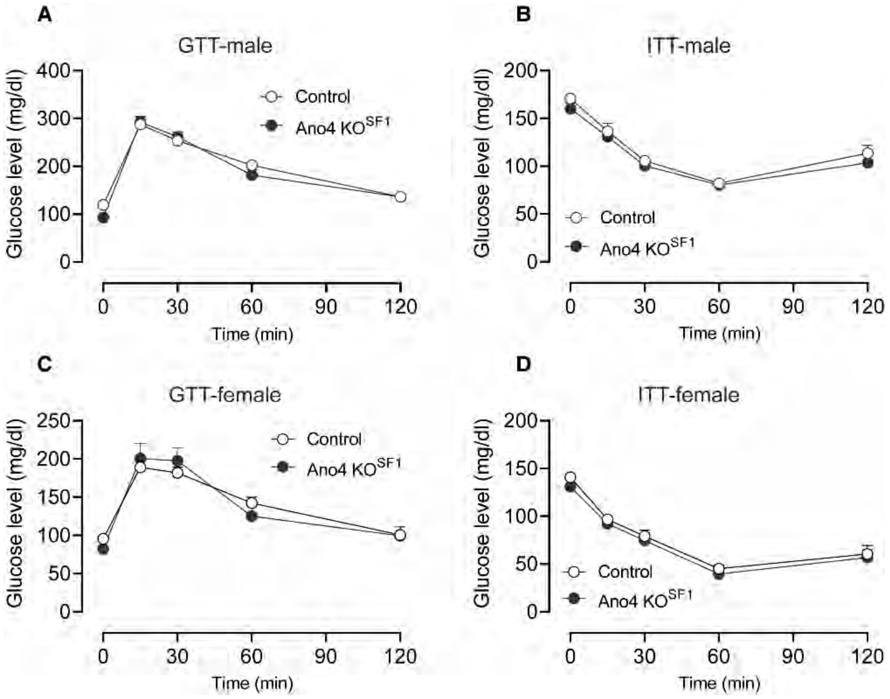
A
GTT-male
Glucose level (mg/dl)
0
100
200
300
400
Control
Ano4 KO^{SF1}
0
30
60
90
120
Time (min)
B
ITT-male
Glucose level (mg/dl)
0
50
100
150
200
Control
Ano4 KO^{SF1}
0
30
60
90
120
Time (min)
C
GTT-female
Glucose level (mg/dl)
0
50
100
150
200
250
Control
Ano4 KO^{SF1}
0
30
60
90
120
Time (min)
D
ITT-female
Glucose level (mg/dl)
0
50
100
150
200
Control
Ano4 KO^{SF1}
0
30
60
90
120
Time (min)

Supplementary Figure 7. Effects of deleting *Ano4* in the VMH^{SF1} neurons on GTT and ITT.

A-**B**. Glucose tolerance and insulin sensitivity tests in male mice (n=10 for Control, and n=7 for Ano4 KO^{SF1}).

C-**D**. Glucose tolerance and insulin sensitivity tests in female mice (n=7 for Control, and n=6 for Ano4 KO^{SF1}).

Data are expressed as mean ± standard errors of the mean. There was no significant difference between Control vs. Ano4 KO^{SF1} mice. (Relative to **Figure 2**)

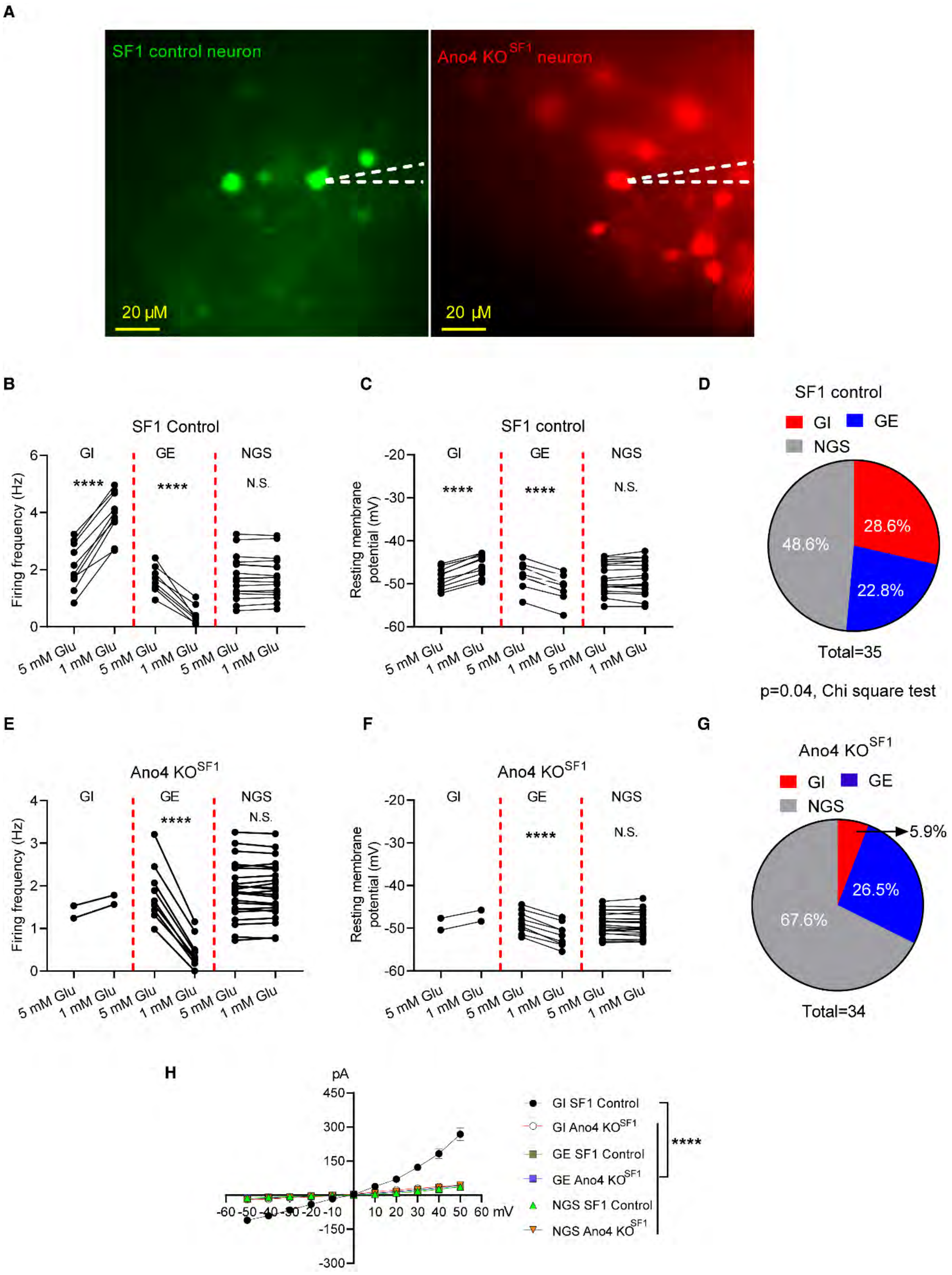

A
SF1 control neuron
Ano4 KO SF1 neuron
20 μM
20 μM
B
C
D
SF1 Control
SF1 control
SF1 control
GI GE NGS
Firing frequency (Hz)
Resting membrane potential (mV)
****
N.S.
5 mM Glu
1 mM Glu
28.6%
22.8%
48.6%
Total=35
p=0.04, Chi square test
E
F
G
Ano4 KO SF1
5.9%
26.5%
67.6%
Total=34
H
pA
mV
GI SF1 Control
GI Ano4 KO SF1
GE SF1 Control
GE Ano4 KO SF1
NGS SF1 Control
NGS Ano4 KO SF1

973

Supplementary Figure 8. Knockout of *Ano4* in the VMH^{SF1} neurons alters the composition of GI neurons.

A. Experimental illustration of a recorded SF1 control neuron and Ano4 KO^{SF1} neuron.

B-D. Firing frequency, resting membrane potential and percentages of GI, GE and NGS neurons in SF1 control neurons under glucose exposure from 5 → 1 mM (n=8 for GE, n=10 for GI and n=17 for NGS).

E-G. Firing frequency, resting membrane potential and percentages of GI, GE and NGS neurons in Ano4 KO^{SF1} neurons under glucose exposure from 5 → 1 mM (n=8 for GE, n=2 for GI, and n=23 for NGS).

H. Ano current detected in GI SF1 control neurons, but not in other groups (n=6 for GI SF1 Control, and n=2 for GI Ano4 KO^{SF1}; n=4 for GE SF1 Control, and n=4 for GI Ano4 KO^{SF1}; and n=5 for GE SF1 Control, and n=5 for GI Ano4 KO^{SF1}).

Data are expressed as mean ± standard errors of the mean. Significant differences between 5 mM glucose and 1 mM glucose are shown as ****$p < 0.0001$ (Paired t-test for **B**, **C, E** and **F;** Two-way ANOVA followed by Bonferroni tests for **H**). Chi square test was used to test significance between **D** and **G**. N.S. represents non-significant. (Relative to **Figure 2**)

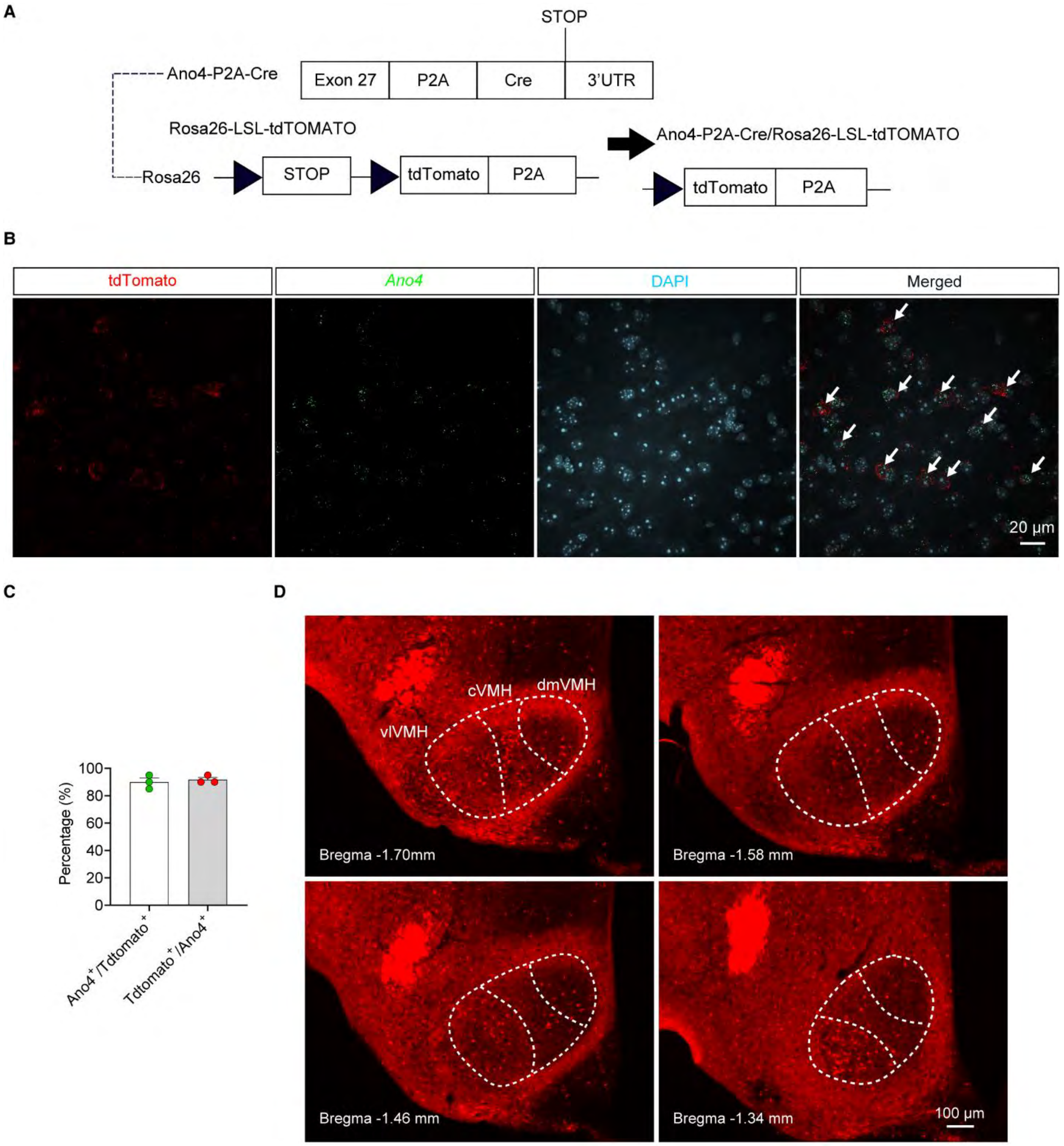
A
STOP
Ano4-P2A-Cre
Exon 27
P2A
Cre
3'UTR
Rosa26-LSL-tdTOMATO
Rosa26
STOP
tdTomato
P2A
Ano4-P2A-Cre/Rosa26-LSL-tdTOMATO
tdTomato
P2A
B
tdTomato
Ano4
DAPI
Merged
20 μm
C
Percentage (%)
100
80
60
40
20
0
Ano4+/Tdtomato+
Tdtomato+/Ano4+
D
cVMH
dmVMH
vlVMH
Bregma -1.70mm
Bregma -1.58 mm
Bregma -1.46 mm
Bregma -1.34 mm
100 μm

992

Supplementary Figure 9. Visualization of Ano4 neurons in the VMH.

A. Strategy of breeding Ano4-P2A-Cre/Rosa26-LSL-tdTOMATO mouse line by crossing Ano4-P2A-Cre mouse line with Rosa26-LSL-tdTOMATO reporter mouse line.

B-C. Co-localization of *Ano4* mRNA with tdTomato mRNA in the VMH of Ano4-P2A-Cre/Rosa26-LSL-tdTOMATO mouse (n=3).

D. Distribution of Ano4 neurons in the VMH of Ano4-P2A-Cre/Rosa26-LSL-tdTOMATO mouse.

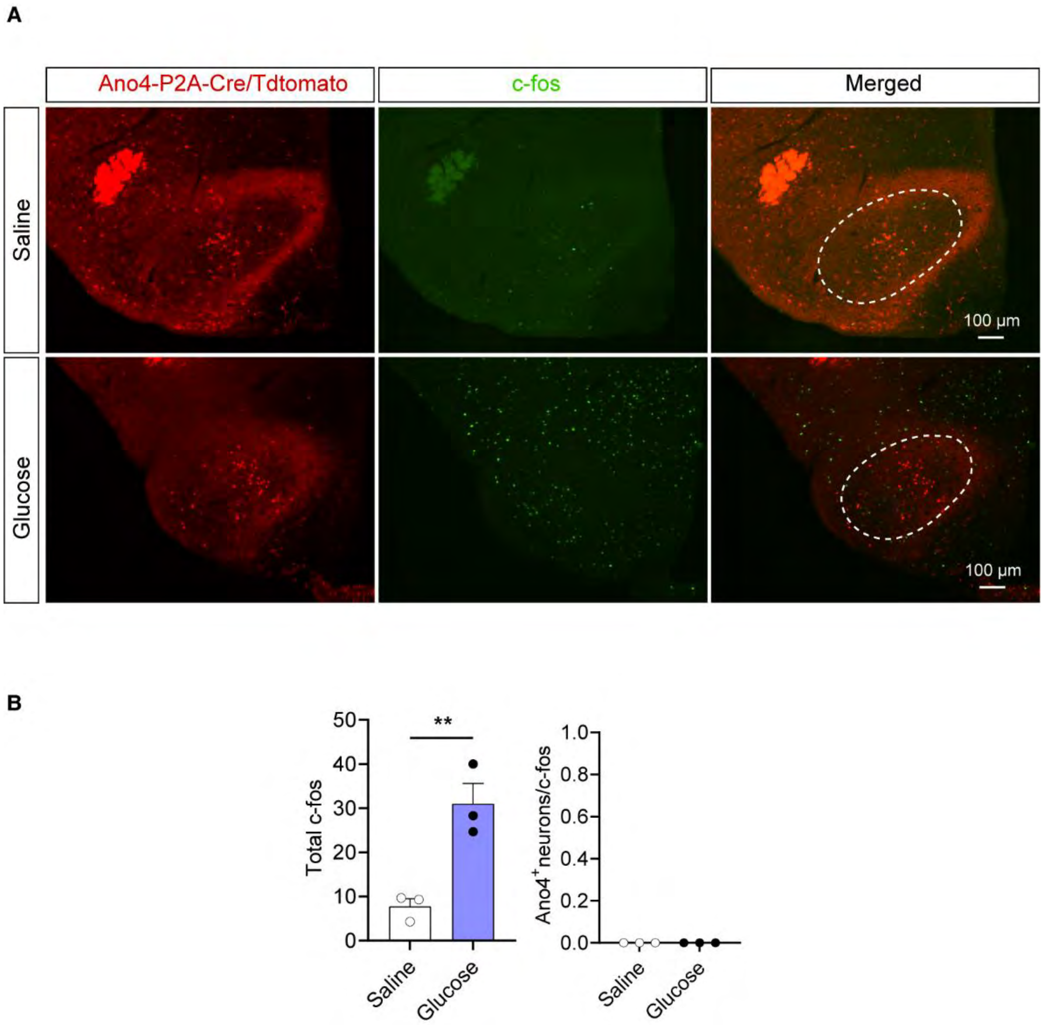
A
Ano4-P2A-Cre/Tdtomato
c-fos
Merged
Saline
Glucose
100 µm
100 µm
B
Total c-fos
**
50
40
30
20
10
0
Saline
Glucose
Ano4⁺neurons/c-fos
1.0
0.8
0.6
0.4
0.2
0.0
Saline
Glucose

1002

Supplementary Figure 10. Glucose-induced c-fos in the VMH did not co-localize with VMH^{Ano4} neurons.

A. Representative images of c-fos staining and Ano4-expressing neurons in the VMH in Ano4-P2A-Cre/Rosa26-LSL-tdTOMATO (male, 8-12 weeks of age) treated with saline (10 ml/kg, i.p.), or glucose (2 g/kg, i.p.).

B. Total c-fos positive cells and Ano4^{+}/c-fos cells in the VMH induced by saline or glucose (n=3 for Saline and Glucose).

Data are expressed as mean ± standard errors of the mean. Significant differences between glucose and saline groups are shown as **p < 0.01 (T-test for **B**).

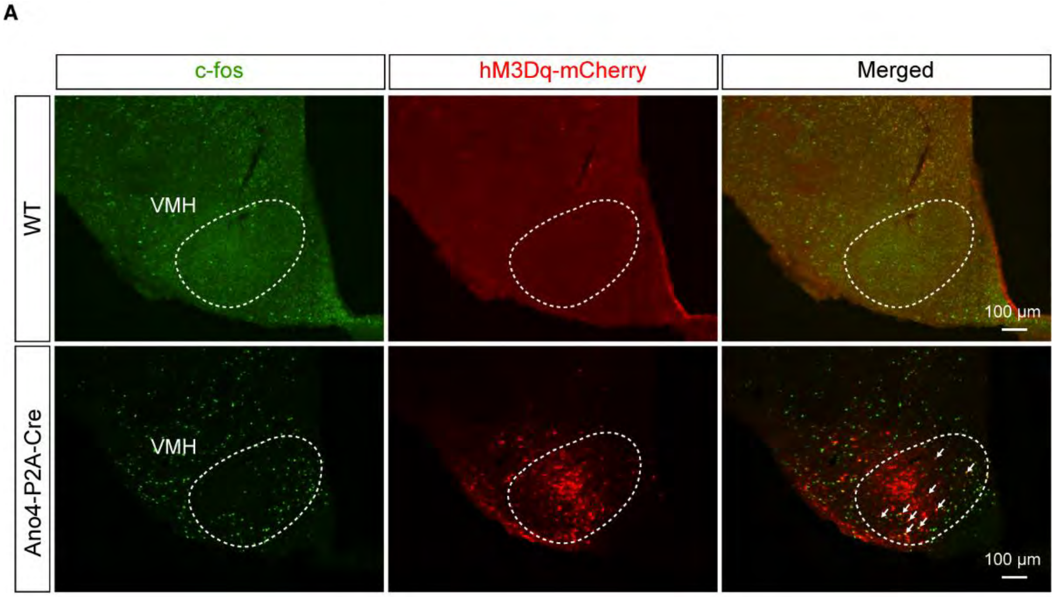
A
c-fos
hM3Dq-mCherry
Merged
WT
VMH
100 μm
Ano4-P2A-Cre
VMH
100 μm

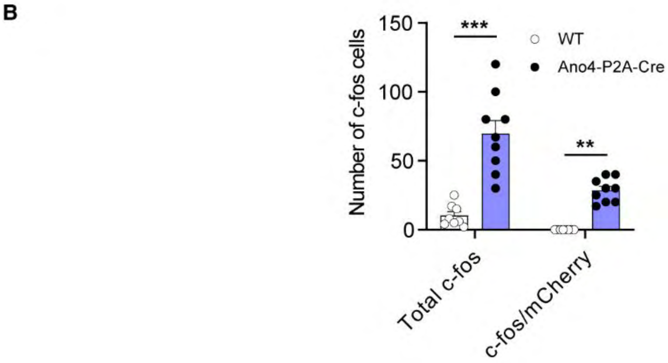
B
Number of c-fos cells
150
100
50
0
***
**
WT
Ano4-P2A-Cre
Total c-fos
c-fos/mCherry

1014

Supplementary Figure 11. Validation of DREADD-mediated activation of VMH^{Ano4} neurons.

A. Representative images of c-fos staining and hM3Dq-mCherry-expressing neurons in the VMH in WT or Ano4-P2A-Cre (male, 12-16 weeks of age) treated with CNO (3 mg/kg, i.p.)

B-C. Total c-fos positive cells and c-fos/mCherry cells in the VMH induced by CNO (n=8 for WT, and n=9 for Ano4-P2A-Cre).

Data are expressed as mean ± standard errors of the mean. Significant differences between glucose and saline groups are shown as $^{**}p < 0.01$, $^{***}p < 0.001$ (T-test for **B**). (Relative to **Figure 4**)

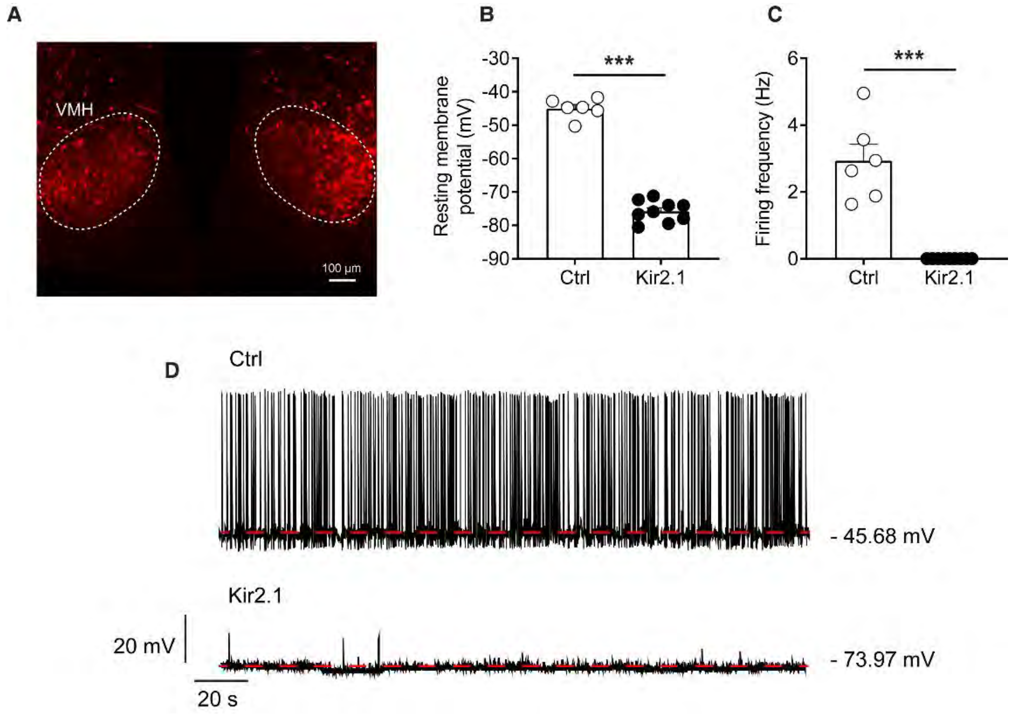
A
VMH
100 µm
B
Resting membrane potential (mV)
-30
-40
-50
-60
-70
-80
-90
***
Ctrl
Kir2.1
C
Firing frequency (Hz)
6
4
2
0
***
Ctrl
Kir2.1
D
Ctrl
- 45.68 mV
Kir2.1
20 mV
- 73.97 mV
20 s

1025

Supplementary Figure 12. Validation of chronic inactivation of VMH^{Ano4} neurons.

A. Representative immunofluorescence images of Kir2.1-P2A-dTOMATO in the VMH in Ano4-P2A-Cre mice.

B-**C**. Quantification of resting membrane potential and firing frequency of VMH^{Ano4} neurons (n=6 for Ctrl, and n=9 for Kir2.1).

D. Representative traces of action potential of a control VMH^{Ano4} neuron and an inactivated VMH^{Ano4} neuron.

Data are expressed as mean ± standard errors of the mean. Significant differences between Ctrl (control) and Kir2.1 are shown as ***$p < 0.001$ (T-test for **B** and **C**). (Relative to **Figure 4**).

1038

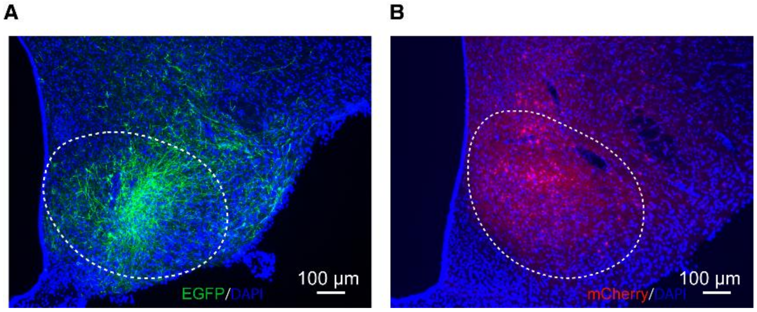

Supplementary Figure 13. Representative immunofluorescent images of hChR2 expression the VMH in Ano4-P2A-Cre mice.

A. VMH^{Ano4} neurons (green color) infected by DIO-hChR2 (H134R)-EFYP with DAPI counter staining.

B. $VMH^{non\text{-}Ano4}$ neurons (red color) infected by DO-hChR2 (H134R)-mCherry with DAPI counter staining. (Relative to **Figure** 5 and **Figure 6**).

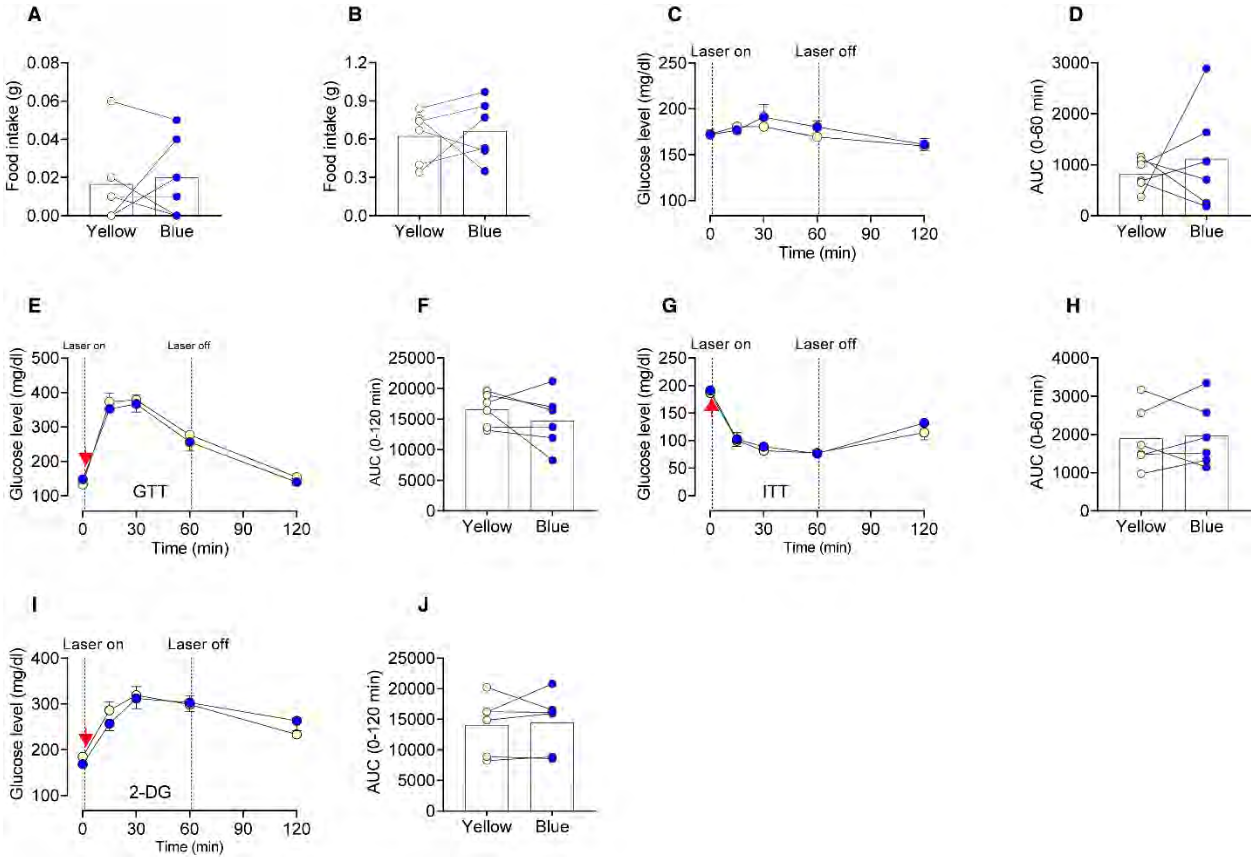

A
B
C
D
Food intake (g)
Yellow
Blue
Laser on
Laser off
Glucose level (mg/dl)
Time (min)
AUC (0-60 min)
E
F
G
H
GTT
AUC (0-120 min)
ITT
I
J
2-DG

1047

Supplementary Figure 14. Blue light or yellow light did not affect food intake or blood glucose levels in control mice.

A-**B**. Food intake in satiated and fasted condition (male, 8-12 weeks of age) (n=6).

C-**F**. Blood glucose levels in a basal state (**C**-**D**), and in glucose tolerance test (**E**-**F**), insulin sensitivity test (**G**-**H**) and 2-DG-induced glucopenia (**I**-**J**) (n=6).

Data are expressed as mean ± standard errors of the mean. There was no significant difference between two groups. Red arrows indicate where glucose (**E**), insulin (**G**) or 2-DG (**I**) was injected.

(Relative to **Figure 5**)

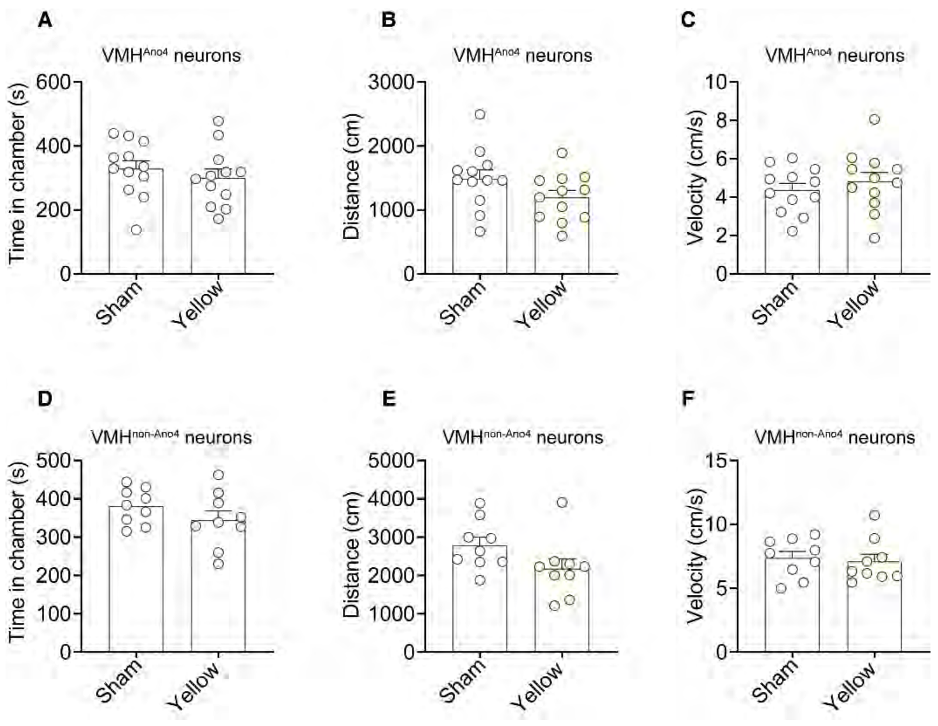

A
VMH^Ano4 neurons
Time in chamber (s)
600
400
200
0
Sham
Yellow
B
VMH^Ano4 neurons
Distance (cm)
3000
2000
1000
0
Sham
Yellow
C
VMH^Ano4 neurons
Velocity (cm/s)
10
8
6
4
2
0
Sham
Yellow
D
VMH^non-Ano4 neurons
Time in chamber (s)
500
400
300
200
100
0
Sham
Yellow
E
VMH^non-Ano4 neurons
Distance (cm)
5000
4000
3000
2000
1000
0
Sham
Yellow
F
VMH^non-Ano4 neurons
Velocity (cm/s)
15
10
5
0
Sham
Yellow

1058

Supplementary Figure 15. Yellow light stimulation of VMH^{Ano4} and VMH$^{non\text{-}Ano4}$ neurons in the VMH did not alter real-time place preference.

A-**C**. Time spent, distance travelled and velocity in each respective chamber for Ano4-P2A-Cre mice (male, 8-12 weeks of age) with injection of AAV2-EF1a-DIO-hChR2 (H134R)-EFYP into the VMH during yellow light stimulation (n=12).

D-**F**. Time spent, and distance travelled and velocity in each respective chamber for Ano4-P2A-Cre mice (male, 8-12 weeks of age) with injection of pAAV-EF1a-DO-hChR2 (H134R)-mCherry into the VMH during yellow light stimulation (n=9).

Data are expressed as mean ± standard errors of the mean. There was no significant difference between sham and yellow light stimulation. (Relative to **Figure 5** and **Figure 6**)

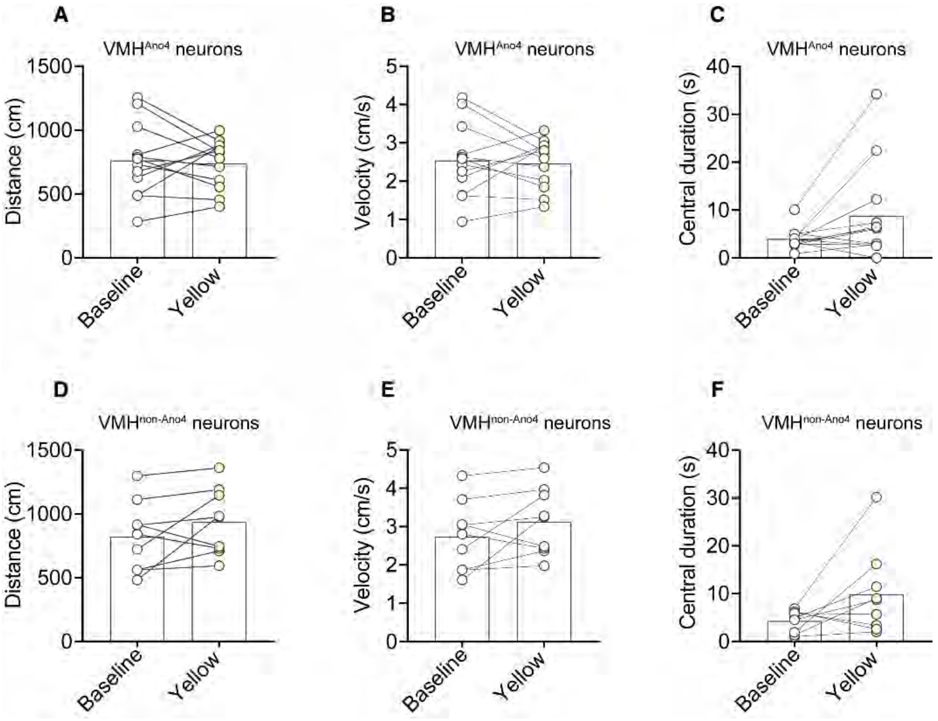
A
VMH^Ano4 neurons
Distance (cm)
1500
1000
500
0
Baseline
Yellow
B
VMH^Ano4 neurons
Velocity (cm/s)
5
4
3
2
1
0
Baseline
Yellow
C
VMH^Ano4 neurons
Central duration (s)
40
30
20
10
0
Baseline
Yellow
D
VMH^non-Ano4 neurons
Distance (cm)
1500
1000
500
0
Baseline
Yellow
E
VMH^non-Ano4 neurons
Velocity (cm/s)
5
4
3
2
1
0
Baseline
Yellow
F
VMH^non-Ano4 neurons
Central duration (s)
40
30
20
10
0
Baseline
Yellow

1071

Supplementary Figure 16. Yellow light stimulation of VMH^{Ano4} and $VMH^{non\text{-}Ano4}$ neurons did not alter locomotor activity.

A-**C**. Distance travelled, velocity and time spent in the center for Ano4-P2A-Cre mice with (male, 8-12 weeks of age) injection of AAV2-EF1a-DIO-hChR2 (H134R)-EFYP into the VMH during yellow light stimulation (n=12).

D-**F**. Distance travelled, velocity and time spent in the center for Ano4-P2A-Cre mice (male, 8-12 weeks of age) with injection of pAAV-EF1a-DO-hChR2 (H134R)-mCherry into the VMH during yellow light stimulation (n=9).

Data are expressed as mean ± standard errors of the mean. There was no significant difference between baseline and yellow light stimulation. (Relative to **Figure 5** and **Figure 6**)

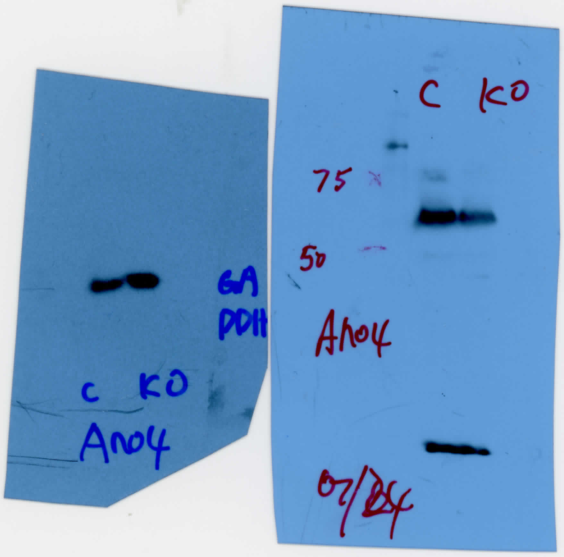

GA
PDH
c KO
Ano4
C KO
75
50
Ano4
